# Agricultural Management and Climatic Change Are the Major Drivers of Biodiversity Change in the UK

**DOI:** 10.1371/journal.pone.0151595

**Published:** 2016-03-23

**Authors:** Fiona Burns, Mark A. Eaton, Kate E. Barlow, Björn C. Beckmann, Tom Brereton, David R. Brooks, Peter M. J. Brown, Nida Al Fulaij, Tony Gent, Ian Henderson, David G. Noble, Mark Parsons, Gary D. Powney, Helen E. Roy, Peter Stroh, Kevin Walker, John W. Wilkinson, Simon R. Wotton, Richard D. Gregory

**Affiliations:** 1 The RSPB Centre for Conservation Science, Sandy, United Kingdom; 2 Bat Conservation Trust, London, United Kingdom; 3 Centre for Ecology & Hydrology, Wallingford, United Kingdom; 4 Butterfly Conservation, Wareham, United Kingdom; 5 Department of AgroEcology, Rothamsted Research, Harpenden, United Kingdom; 6 Anglia Ruskin University, Cambridge, United Kingdom; 7 People’s Trust for Endangered Species, London, United Kingdom; 8 Amphibian and Reptile Conservation, Bournemouth, United Kingdom; 9 British Trust for Ornithology, Thetford, United Kingdom; 10 Botanical Society of Britain and Ireland, Bristol, United Kingdom; Aberystwyth University, UNITED KINGDOM

## Abstract

Action to reduce anthropogenic impact on the environment and species within it will be most effective when targeted towards activities that have the greatest impact on biodiversity. To do this effectively we need to better understand the relative importance of different activities and how they drive changes in species’ populations. Here, we present a novel, flexible framework that reviews evidence for the relative importance of these drivers of change and uses it to explain recent alterations in species’ populations. We review drivers of change across four hundred species sampled from a broad range of taxonomic groups in the UK. We found that species’ population change (~1970–2012) has been most strongly impacted by intensive management of agricultural land and by climatic change. The impact of the former was primarily deleterious, whereas the impact of climatic change to date has been more mixed. Findings were similar across the three major taxonomic groups assessed (insects, vascular plants and vertebrates). In general, the way a habitat was managed had a greater impact than changes in its extent, which accords with the relatively small changes in the areas occupied by different habitats during our study period, compared to substantial changes in habitat management. Of the drivers classified as conservation measures, low-intensity management of agricultural land and habitat creation had the greatest impact. Our framework could be used to assess the relative importance of drivers at a range of scales to better inform our policy and management decisions. Furthermore, by scoring the quality of evidence, this framework helps us identify research gaps and needs.

## Introduction

Despite the efforts of conservationists, and widespread public support for conservation action, biodiversity continues to be lost and is predicted to decline further by 2020 [1,2]. This loss has been observed at many scales, enabled by a growth in our ability to measure change. The loss of biodiversity in recent decades e.g. as reported in the UK [3] is caused by an imbalance between increasing pressures on nature on the one hand [2] and insufficient policy and management responses on the other [1,2]. There are many reasons to conserve biodiversity and attempt to reverse changes observed, ranging from an intrinsic moral duty of care, to a utilitarian desire to maximise benefits to humans [4]. Regardless of the motivation, responses to the decline of biodiversity must be informed by an understanding of the key factors affecting change (‘drivers of change’) in order to make effective use of the limited resources available.

At a global scale, the most important direct drivers of biodiversity loss and ecosystem service changes are considered to be habitat change and degradation, climatic change, invasive species (native and non-native), overexploitation, and pollution [5]. Within the UK, the National Ecosystem Assessment (NEA; [6]) combined expert opinion with the best available information, and suggested that since the 1940s habitat change, pollution and nutrient enrichment were the major drivers of change, that overexploitation and climatic change were important drivers of change, and invasive species a moderate driver of change. It also suggested that overexploitation, invasive species and especially climatic change would have ongoing impacts on habitat extent and condition.

Whilst the NEA has been influential in the UK, its broad-brush approach makes it difficult to translate the findings into detailed conservation planning and action, and a full review of the drivers of changes in species’ population status was beyond its scope [6]. A considerable body of ecological research has focussed on understanding the reasons underlying recent population trends for individual species or habitats (e.g. [7–10]), but it is difficult to generalise from these.

We extend the quantitative/qualitative framework introduced by the NEA to assess the drivers of change in a large sample of UK species and taxonomic groups. We are able to take advantage of a recent comprehensive assessment of species change in the UK, *The State of Nature* report [3]. The report summarised quantitative trends in abundance and distribution in the UK over the last 50 years for over 3000 species. It found that species declines outweighed species increases, and that half the species assessed showed strong changes. In this paper we explore the relative importance of the factors that have driven the species’ changes reported in the UK [3], examining both those that have negative and positive impacts on populations. We examined a sample of four hundred species of vertebrates, invertebrates and vascular plants.

We used our review to address the following questions: Which drivers of change have had the greatest impact (assessed as Absolute, Negative and Positive impact) on individual species’ population trends from 1970 to 2012? How do these drivers vary between different taxonomic groups? What has been the overall impact of drivers that are predominantly the result of conservation action? And what are the most important gaps in our knowledge? Our assessment took a species-focussed approach, dictated by the availability of suitable evidence. It should be remembered, however, that drivers of change act broadly across habitats and landscapes, and conservation responses to counter drivers of biodiversity loss may similarly be delivered using broad scale approaches.

## Materials and Methods

**Fig 1:**
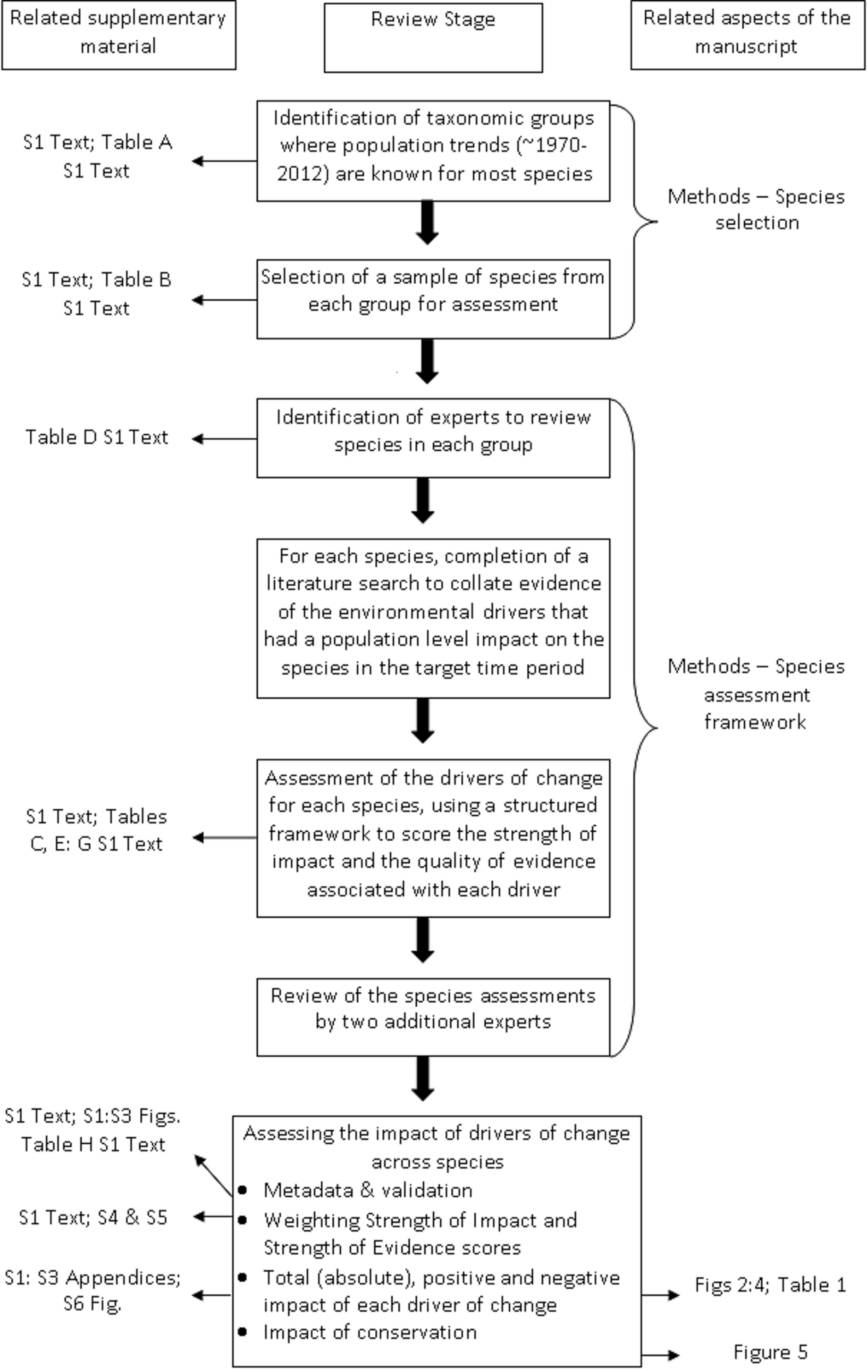
Overview of the review process. The relevant sections of the manuscript and supplementary material to go to for further information are shown in the right-hand and left-hand columns respectively.

A summary of the review process and where to find information on each stage is shown in Fig 1.

### Species selection

Our review covers the taxonomic groups and species of terrestrial and freshwater habitats in the UK for which trends in population abundance, frequency of occurrence, or range were available for the majority of species. We focussed on groups and species where the analysis methods used to derive trends had been peer-reviewed (Table A in S1 Text). We refer to these trends, in whichever metric, as ‘population change’ hereafter. We aimed to assess a sufficient number of species within each taxonomic group so that the sample was broadly representative of the full group: a minimum of 20 species per group, which for many groups (mammals (excluding bats), bats, amphibians, reptiles and Orthoptera) meant assessing all species with available population trends, and for others (ladybirds, Odonata) meant selecting a sample of ~20 species. For more species-rich groups (birds, butterflies, moths and vascular plants), sample sizes were based on a simple randomisation test (S1 Text; Table B S1 Text).

### Species assessment framework

The drivers of population change were assessed by researchers with expertise on each taxonomic group using a structured framework to ensure a consistent approach was used throughout (Fig 1). The co-authors are all technical staff from nature conservation charities and research organisations; one of the co-authors took charge of the review process for each taxonomic group, allocating the assessments between themselves and colleagues and peers from other research establishments. These experts gathered the available scientific peer-reviewed and grey literature, and used this alongside their expert opinion to consider all the relevant drivers of change from a set list (Table C in S1 Text), and to identify those drivers for which there was evidence of impact on the species in question. A set literature search strategy was followed. This determined standardised search terms (Google scholar: (“latin name” OR”English name”) driver change (UK OR Europe)) and suggested that assessors investigated the first three pages of results (30) as a minimum.

Given that each species assessment required interpretation of the literature, all assessments were open to subjectivity. To address this issue, we aimed to get at least three experts to contribute to each species assessment (Table D in S1 Text). This condition was met for all groups with the exception of moths, ladybirds, vascular plants and four mammal species, for which only two experts were available. For each species, one person conducted the initial assessment and circulated the draft results to two or more others for comment and refinement. Comments were shared between those involved until all experts were in agreement with each assessment. This process was designed to ensure that all assessments within a taxonomic group were consistent in how the drivers were identified and how the strength of, and evidence for, that impact were scored. The review process was open and the identity of all experts known, including which species assessments they had completed or reviewed.

We used a modified version of lists of potential drivers used to assess threats for IUCN Red listed species, and for species on the annexes of the EU Habitats and Birds Directives ([11] Table C in S1 Text). Since our review covers all drivers of change, not just threats, we added categories to describe drivers of positive change, including conservation measures. The list had two hierarchical levels of detail, ‘Broad drivers’, each of which contained one or more ‘Specific drivers’. The list included categories for ‘No known drivers of change’ (the reasons for recent population change are entirely unknown), ‘No drivers of change’ (evidence exists to suggest that no drivers of change had a population level impact on the species) and ‘Unknown driver of change’ (known drivers only partially explain the observed species population change). Experts used the category ‘Other’ in cases where the driver did not fit those available. In addition, experts were asked to identify when a driver acted upon a species through changing levels of habitat connectivity and/or heterogeneity. We considered impacts due to a decrease, or low levels of a driver (e.g. decreasing or low levels of water pollution) separately from those due to an increase or high levels of the driver (e.g. increasing or high levels of water pollution). We scored each driver as having a high or low level of anthropogenic impact and whether it was related to conservation action (Table C in S1 Text). Each driver was scored on a 1 to 12 scale to reflect the Strength of Impact on the species (Table E in S1 Text), and also for the Strength of the Evidence upon which the assessment was based (Table F in S1 Text). The Strength of Impact score was given a sign to indicate a negative or positive impact on the population of the species in question. The use of an interval scale meant that, for example, two drivers assessed with a score of six had the same total impact as a single driver scored as 12. A small number of drivers were assessed as having ‘unknown’ strength of impact and were given an impact score of 1, on the assumption that if there was no knowledge on the strength of impact, it was most likely to be trivial. Experts were asked to include comments explaining the drivers and scores selected. To help our assessors, we collated published information on the change in environmental parameters related to each driver of change over the study period (Table G in S1 Text).

### Assessing the impact of drivers of change across species

#### Metadata and validation

As described above, we took steps to ensure that assessments were undertaken consistently between the different taxonomic groups and carried out validation to assess any such variation (S1 Text; Table H S1 Text; S1–S3 Figs). We excluded from the analysis species for which the drivers of change were entirely unknown and those that were assessed as having no drivers of change. The driver category ‘Other’ was used only on seven occasions and described such a broad spectrum of drivers as to preclude synthesis, therefore these instances were removed from further analysis.

#### Weighting Strength of Impact and Strength of Evidence scores

A different number and proportion of species were assessed in each taxonomic group. We therefore weighted each *Strength of Impact* score as if we had assessed the same number of species of each higher taxonomic group (vascular plants, vertebrates and invertebrates) (Eq 1), so each higher group has equal weight in the results we present. We also looked at weighting Strength of Impact scores in other ways (S1 Text; S4 Fig). For clarity, and because the *Strength of Evidence* score had little impact on the results (S1 Text; S5 Fig), we present results using all levels of evidence.

$\;WI_{h}=I_{h}.\frac{N}{H{.N}_{h}}$ (Eq 1; WI = weighted Strength of Impact score; I = Strength of Impact scores; H = number of higher taxonomic groups; and N = number of species. Subscript letters denote that the parameter is specific to the target higher taxonomic group (h))

#### Absolute, positive and negative impact of each driver of change

We summarised our data at the level of taxonomic group, higher taxonomic group (insects, vertebrates and vascular plants) and across all species (Table A in S1 Text) using the weighted Strength of Impact scores as described above. For each broad driver of change we calculated its absolute impact (the absolute sum of all weighted Strength of Impact scores allocated to that driver), its positive impact (the sum of all the positive weighted Strength of Impact scores allocated to that driver), and its negative impact (the sum of all the negative weighted Strength of Impact scores allocated to that driver), treating the two directions of the driver representing high and low levels of anthropogenic impact separately. In all cases, we expressed the summed weighted Strength of Impact scores for each driver as a percentage of the total across all drivers of change.

#### Impact of Conservation

To investigate the impact of conservation action we summarised our data as described above, for the subset of drivers primarily associated with conservation measures (Table C in S1 Text). We also summarised the impact of drivers associated with changes in habitat connectivity and heterogeneity. Finally, we assessed whether the subset of the species assessed that are listed as conservation priorities by one or more of the UK countries [12], experienced a greater benefit from conservation impact compared to those that are not conservation priorities. All data manipulation and analysis was carried out in R 3.2.1[13].

## Results

### Metadata and validation

Of the 398 species selected for assessment, 48 were assessed as having no known drivers of change (mostly vascular plants or ladybirds) and 18 as having no drivers of change (mostly vascular plants and moths), and as such were excluded from further assessment, leaving 332 species.

The majority of evidence supporting the review was judged to be of low quality, with six out of every ten scores given for strength of evidence being a four or lower (S1 Appendix). Evidence quality had little impact on the overall conclusions (S5 Fig), however the variation in strength of evidence across taxonomic groups and drivers of change can be used to identify priorities for future research (S1 Fig and S1 Appendix).

### Absolute positive and negative impact of each driver of change

**Fig 2:**
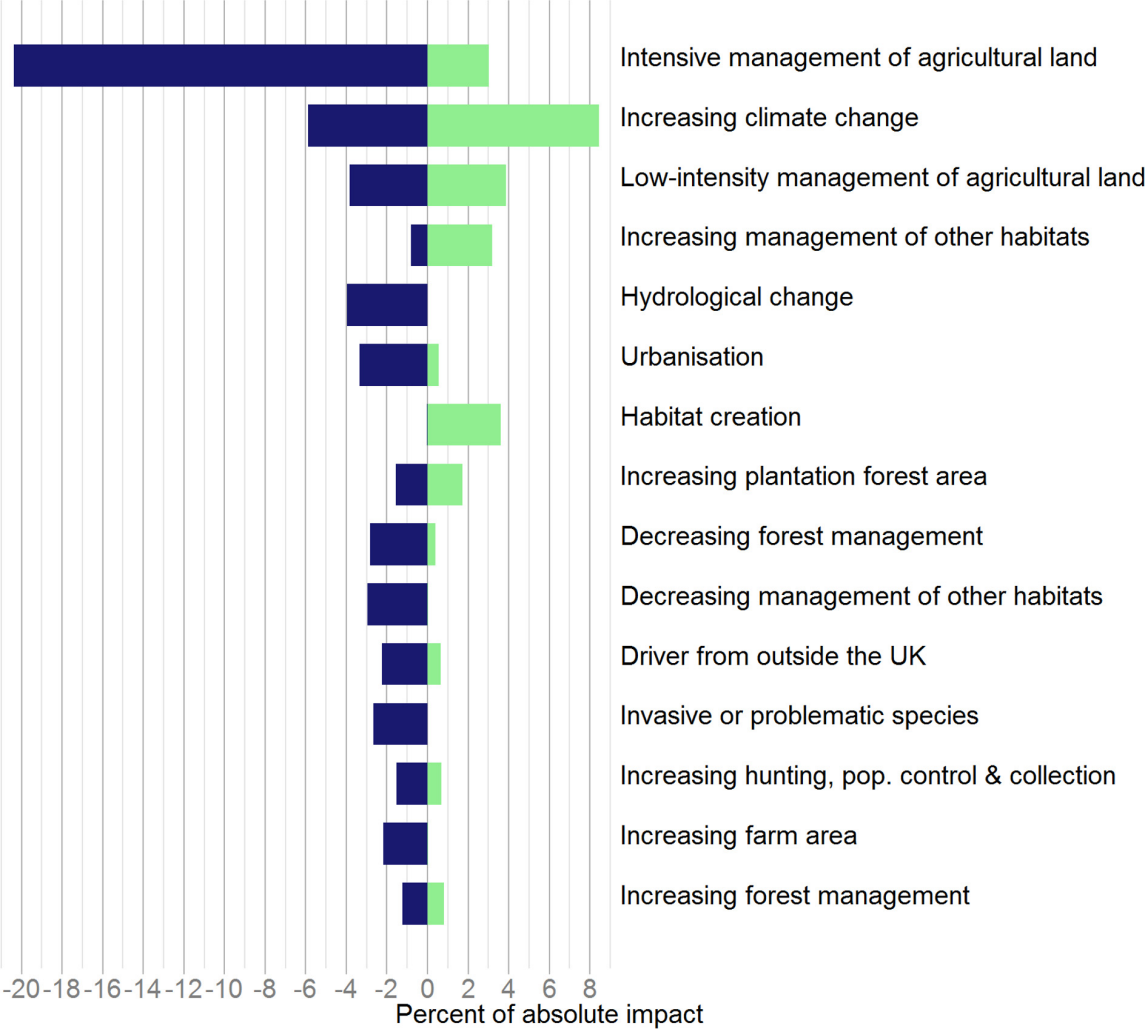
The most important broad drivers of species’ population changes, 1970–2012. Positive (green) and negative (blue) impact for each broad driver of change accounting for two percent or more of the total in absolute terms, ordered by absolute impact. Results are presented using all strengths of evidence available and weighting species in the three major taxonomic groups equally (insects, plants and vertebrates).

In terms of broad drivers of change, intensive management of agricultural land accounted for the largest percentage of both absolute impact, and negative impact, on species, 23 [-20 | +3] (Fig 2; Table 1; S1 Appendix). Climatic change accounted for the second largest percentage of impact, 14 [-6 | +8], though its impact on species trends was more balanced between positive and negative, and thus was the largest positive impact. Overall, across all drivers, two thirds of the impact was negative and one third positive (Table 1). Of the negative impacts, four fifths were classified as high or increasing anthropogenic impact on our environment, but others were due to low or decreasing impact: this was largely accounted for by lack or declining intensity of management of different habitat types.

**Table 1:** Broad drivers of change on UK biodiversity, 1970–2012, that accounted for two percent of absolute impact or greater. Results are presented using all strengths of evidence available and weighting species in the three higher taxonomic groups equally (insects, plants and vertebrates). Full results are given in S1 Appendix.

Broad driver, with direction	Impact score^1^				Number of species impacted (number excluding low quality evidence given in brackets)
Intensive management of agricultural land	23	[-20	|	+3]	171 (64)
Increasing climate change	14	[-6	|	+8]	152 (67)
Low-intensity management of agricultural land^3^	8	[-4	|	+4]	61 (44)
Hydrological change^4^	4	[-4	|	+0]	49 (10)
Increasing management of other habitats	4	[-1	|	+3]	37 (17)
Decreasing forest management	3	[-3	|	+0]	56 (11)
Urbanisation	4	[-3	|	+1]	43 (16)
Habitat creation	4	[-0	|	+4]	38 (11)
Increasing plantation forest area^4^	3	[-2	|	+2]	37 (11)
Decreasing management of other habitats	3	[-3	|	+0]	32 (9)
Increasing farm area	2	[-2	|	+0]	40 (5)
Driver from outside the UK	3	[-2	|	+1]	21 (12)
Invasive non-native species or problematic species	3	[-3	|	+0]	30 (15)
Increasing air pollution	2	[-2	|	+0]	23 (9)
Increasing hunting, population control & collection	2	[-2	|	+1]	19 (10)
Increasing forest management	2	[-1	|	+1]	20 (8)
Decreasing hunting, population control & collection	2	[-0	|	+1]	11 (9)
Decreasing human disturbance	2	[-0	|	+2]	11 (2)
**Total Impact^2^**	**100**	**[-65**	**|**	**+34]**	

1: Impact scores are expressed as a percent of absolute impact across all drivers and species. The absolute impact is given for each driver, followed by a break down into negative and positive impacts

2: Impact scores presented here do not sum to 100 as drivers with <2% impacts were excluded

3: Broad driver where the supporting evidence is stronger than average

4: Broad driver where the supporting evidence is weaker than average.

**Fig 3:**
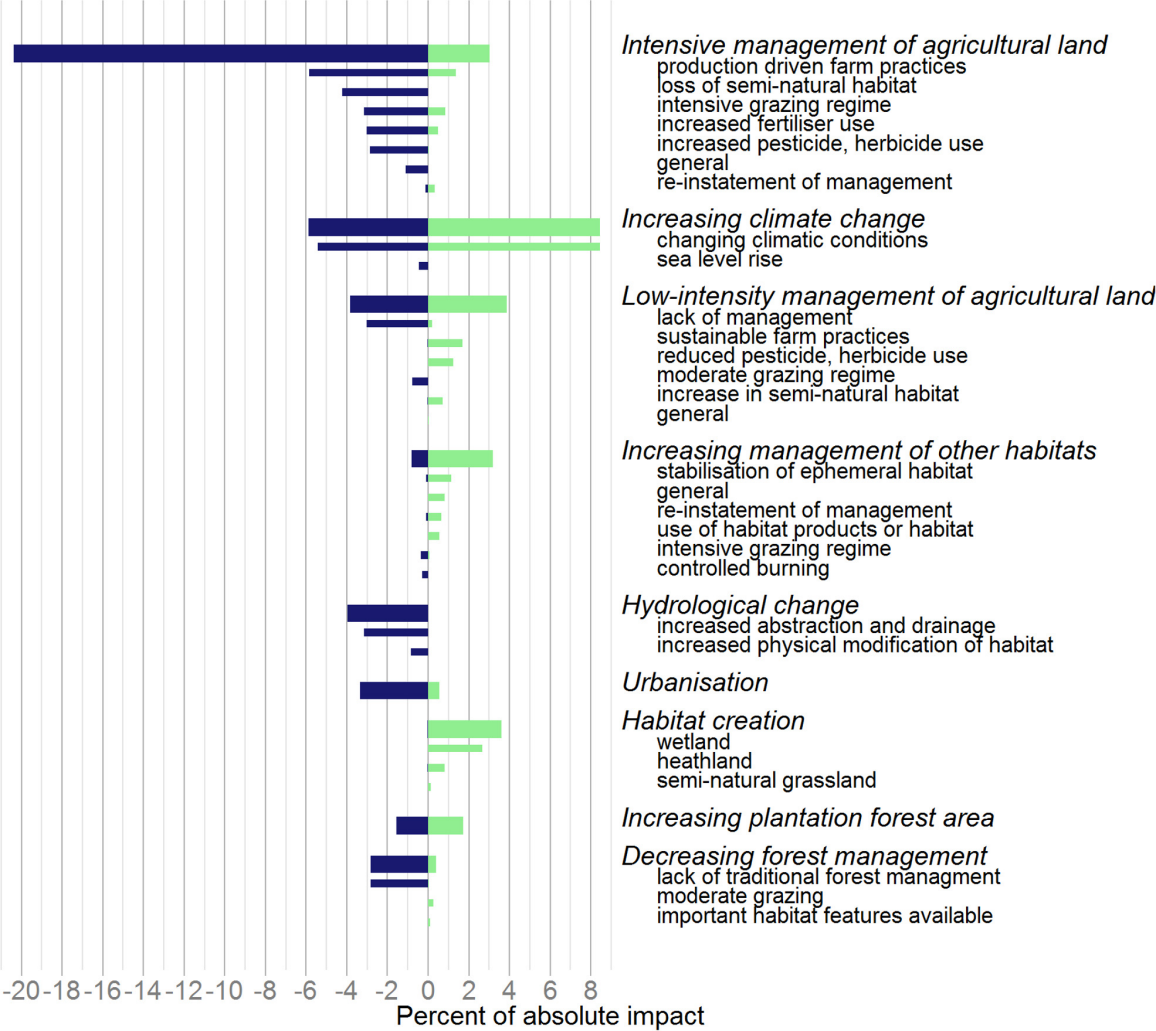
The most important broad drivers of species’ population changes, 1970–2012, showing constituent specific drivers. Positive (green) and negative (blue) impact for each broad driver of change accounting for three percent or more of the total in absolute terms, ordered by absolute impact. Specific drivers (narrow bars) are listed under their associated broad driver (broad bars, italicised text); the impact of specific drivers sum to the total for the broad driver in each case. Results are presented using all strengths of evidence available and weighting species in the three major taxonomic groups equally (insects, plants and vertebrates).

The specific drivers illustrate the results in greater detail (Fig 3; S1 Appendix). These show, for example, that the impact of intensive management of agricultural land was spread across a range of specific drivers including production-driven farm practices (e.g. timing of sowing or mowing), decrease in semi-natural habitat in farmland (e.g. hedgerows or ponds), as well as the application of agri-chemicals.

**Fig 4:**
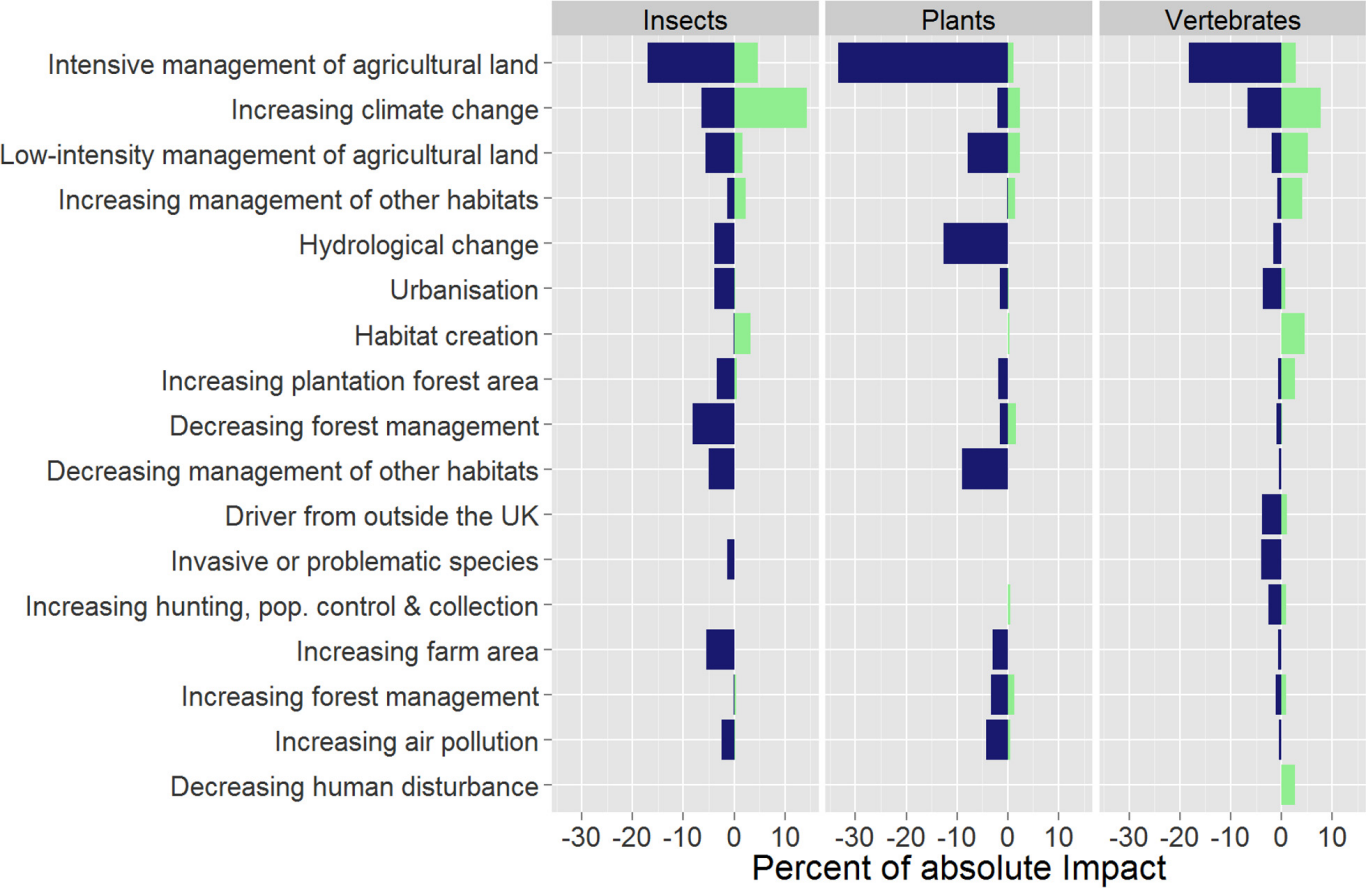
The most important broad drivers of species’ population changes, 1970–2012, by higher taxonomic group. Positive (light green) and negative (dark blue) impact for each broad driver of change accounting for two percent or more of the total in absolute terms, ordered by absolute impact; by higher taxonomic group. Impact is shown as a percentage of the impact on that group, i.e. absolute impact sums to 100 for each of the three groups. Results are presented using all strength of evidence available.

The proportion of absolute impact attributed to vertebrates (0.6) was higher than for insects (0.24) or plants (0.16), presumably reflecting the level of knowledge of these groups, and a bias in research and conservation action targeted towards them. Given the difference between the three higher taxonomic groups it was useful to investigate the relative importance of broad drivers within each group separately (Fig 4; S2 Appendix). The patterns were similar within each group e.g. intensive management of agricultural land had the biggest impact on all three groups, with a similar ratio of negative to positive impacts. There were notable exceptions; climatic change was the second biggest impact on vertebrates and insects, but only the seventh biggest impact on plants. Some drivers of change were primarily associated with one or two higher taxonomic groups, for example, the negative impact of hydrological change was relatively greater for vascular plants, whereas the positive impact of habitat creation was relatively lower for vascular plants.

Similar patterns of evenness of each driver’s impact were present when the eleven taxonomic groups were compared, although variation was generally higher (S6 Fig; S3 Appendix). Intensive management of agricultural land had the biggest impact on six of the eleven taxonomic groups and climate change was the first or second largest impact for seven of the eleven groups. For a small number of groups an alternative driver dominated impact, for example, an invasive non-native species had the single biggest impact on ladybirds.

Several broad drivers of change related to an increase or decrease in the extent of a habitat type, for example increasing farm area, or habitat creation. For each of these there must be a complementary increase or decrease in another habitat type, which experts noted where possible. The most complete information was available for increasing farm area, with around a third of instances linked to a complementary decrease in the area of heathland or bog, semi-natural grassland and wetlands respectively. Decreases in habitat connectivity and heterogeneity accounted for a moderate percentage of absolute impact on species 10 [-10 | +0], whereas efforts to increase connectivity and heterogeneity accounted for a much smaller percentage 2 [-0 | +2]. It is important to note that the lack of effect of this and other such interventions may be related to the levels of interventions, and/or the quality of those interventions, rather than an ineffectiveness *per se*. Decreases in habitat connectivity and heterogeneity were primarily associated with intensive management of agricultural land and decreasing management of forest.

### Impact of Conservation

**Fig 5:**
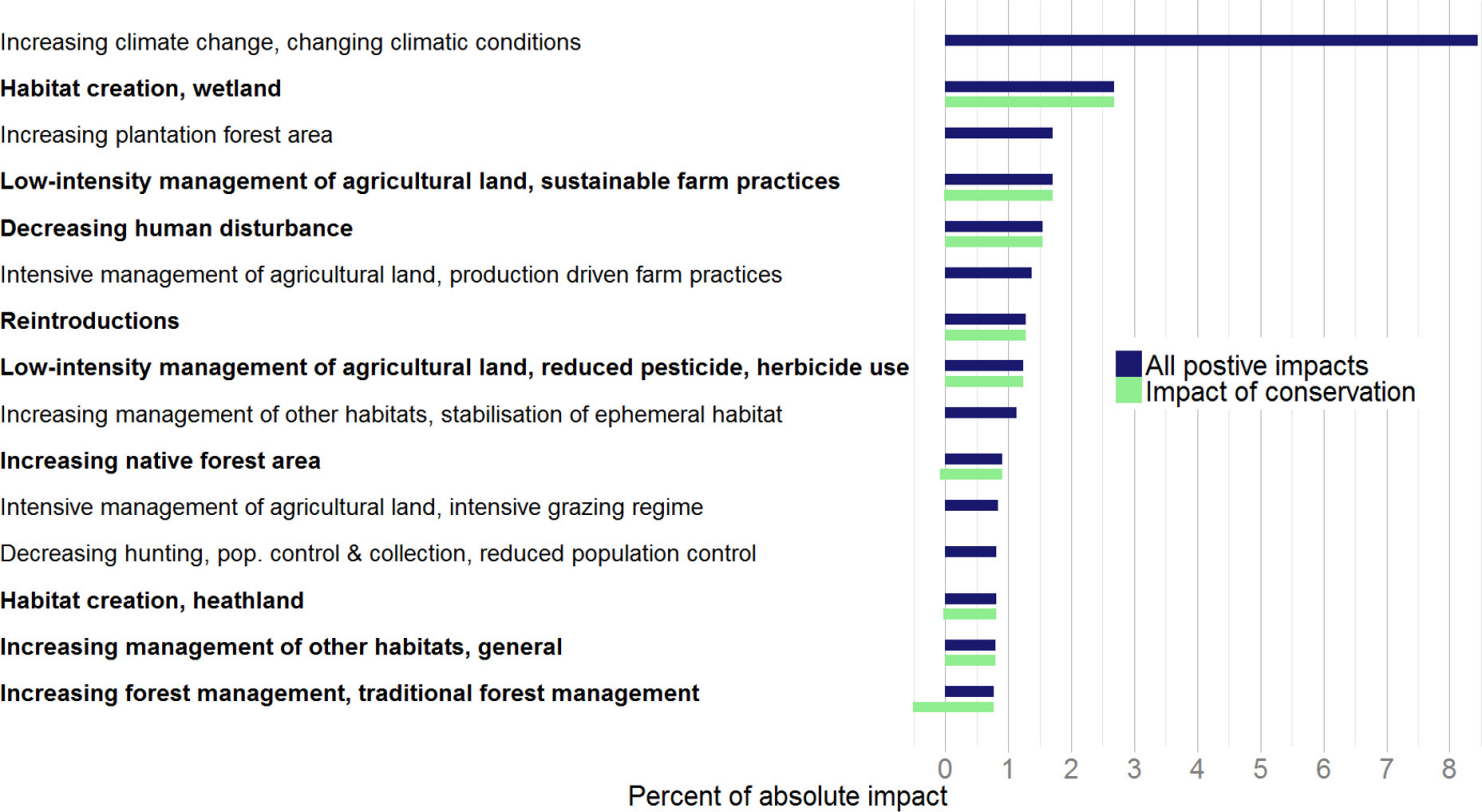
Impact of specific drivers classified as conservation measures in relation to all positive impacts on UK species. Specific drivers of change with positive impacts accounting for at least 0.75% of absolute impact, showing the positive impact, including conservation and non-conservation actions (dark blue) and the impact of conservation alone (light green), in the latter case both positive and negative impacts are shown. Specific drivers categorised as conservation actions are shown in bold.

A subset of drivers with conservation action (Table C in S1 Text) accounted for a substantial percentage of absolute impact 19 [-2 | +17], the majority of which was positive (Fig 5; S1 Appendix). The most important broad drivers categorised as conservation actions were low-intensity management of agricultural land, 5 [-1 | +4] and habitat creation, 4 [-0 | +4]. Within these broad drivers, the specific drivers of sustainable farm practice and creation of wetlands had the biggest impacts. The proportion of impact from actions attributed to conservation was higher for vertebrates (0.71) than for insects (0.18) and vascular plants (0.11) (S2 Appendix). Priority species are those that have been listed as being of conservation concern or importance in the UK and as such they are often the focus of specific, targeted conservation action [12]. Around a third of the species reviewed are listed as priority species (116 out of 332), however, they accounted for a much greater proportion of the impact assessed for drivers categorised as conservation (0.62).

## Discussion

### Overview

In this study we use a new framework to assess the drivers of population change in UK biodiversity and find that agricultural management and climatic change were the two dominant drivers of biodiversity change over the last 40 years. Our framework is novel in that it considers drivers of change in a comprehensive way and is not an assessment of threats alone [14]. We follow the approach of the NEA [6] but importantly, we score both the strength of evidence and strength of impacts to understand the emerging patterns. We have tested for bias (S1 Text), considered different approaches to weighting our findings across taxonomic groups, and treatment of evidence of varying quality to investigate the robustness of our results. Nonetheless, we believe our assessment of the main drivers of species change in the UK is robust and our findings echo those of the NEA [6].

The late 20th century marked a period of great change in the fauna and flora of the British Isles [15]. Much effort has been directed towards understanding the changes in the environment underpinning this change. Here, we reviewed this evidence to assess the relative importance of environmental changes, or drivers of change, across taxonomic groups. Our findings quantify the predominantly negative impact of intensive management of agricultural land upon the UK’s biodiversity in recent decades (1970–2012), as well as the negative impact of hydrological change and urbanisation. Declines in management of farmland (e.g. abandonment), forest and other habitats, also had a primarily negative impact on biodiversity within our sample. Notably, the second largest negative impact was from climatic change, but the magnitude of the positive impact of this driver was similar, and by far the largest positive impact upon species. Other drivers having a substantial positive impact included low-intensity management of agricultural land, and habitat creation.

Our study period starts at a time when the UK’s biodiversity was already at a much altered baseline; there is extensive (primarily qualitative) evidence for huge change, mainly loss, of habitats and biodiversity in the UK stretching back many hundreds of years. This biodiversity loss was driven largely by gross habitat change, when semi-natural habitats such as grasslands and heathlands were converted to enclosed farmland and plantation forestry [6,16]. The role of habitat management as the most significant driver over recent decades should be viewed against a backcloth of prior habitat loss.

### Knowledge gaps

By scoring the quality of evidence for the impact of each driver we were able to identify key knowledge gaps warranting further research. We had stronger evidence and more impacts cited for vertebrate species compared to insects or plants. The strength of evidence available to assess the drivers of change in moth species was substantially lower than that available for other groups (S1 Fig). Given that total abundance of macro-moths declined by 28% between 1968 and 2007 [17] and investigating this decline was listed as one of the top 100 questions for conservation science [18] further research is required (e.g. [17,19]). Across all taxa, one in ten species selected for review had no evidence available to assign drivers of change: these species should be targets for future research. Since we were only able to review taxonomic groups where a substantial proportion of species had known population trends, we omitted considerable sections of biodiversity, including fungi, bryophytes and non-insect invertebrates.

A key limitation of the evidence available to date is that, excluding a few notable exceptions [19–21], drivers of change have been investigated in isolation, and little is known about how they interact [22]. It is clear that the impact of multiple changes in the environment will not necessarily be additive [23], and they could interact in complex ways, making it difficult to design targeted conservation management [24]. For example, the interaction of climatic warming and increased nitrogen deposition is thought to have resulted in microclimatic cooling in certain areas, which has had a negative impact on several insect species [7,25].

### Strengths and weaknesses of our methodological framework

Our review attempted to use consistent methods across a range of taxonomic groups and therefore allows overall conclusions to be drawn. The proportion of positive and negative impacts match well with observed species declines and increases [3], and the net sum of the Strength of Impact scores for the drivers of change listed against each species correlates significantly with the observed population change (S3 Fig). The neutral standpoint of the review is also a strength, as whatever change humans make to the environment there will be a mix of positive and negative biological responses.

Our assessment used impact upon individual species as its basis, and did not consider species population or range size (e.g. by weighting to increase the value of common or widespread species in our analysis). If these were also considered, it is likely that our findings would change to some degree. In particular, it is likely that changes in agricultural management may have had an even greater impact, given the large proportion of UK land area that is farmed (40% is enclosed farmland, with a further 35% being farmed in some way [6]). Despite the steps taken to minimise variation in how different experts used the framework, it is unlikely that we reduced this to zero: for example, where stronger evidence was available experts may have felt more confident in giving high scores for strength of impact, which may have influenced the more detailed aspects of our conclusions.

The list of drivers of change used in the review was chosen to maximise the consistency of our review with similar assessments [11]. Nevertheless, its structure is imperfect, encompassing a mix of habitat-focussed drivers (e.g. farm management) and issue-based drivers (e.g. hydrological change). Therefore some things that derive from the same root cause are split between different categories of driver. For example, the true impact of fertiliser use could be described by a combination of fertiliser use category in agricultural management, plus the impact of nitrogenous air pollution and nitrogenous water pollution caused by fertiliser drift, in the air and water pollution categories respectively; taken together these are an important driver of change. Finally, we reviewed the drivers of change on species over a relatively long time period, due to the time taken to observe change in species and to research the underlying causes of this. However, it does mean that it is difficult to draw robust conclusions about how these impacts have changed more recently or how they are likely to change in the future.

### Intensive management of agricultural land

Agricultural management has changed markedly over the period of our review. This includes practices used to increase productivity, such as a change from spring- to autumn-sown crops and changes to the structure of habitats such as declines in the extent of hedgerows and the number of farm ponds [26]. These changes have driven changes in a wide range of taxonomic groups (e.g. [27–29]). An important finding from this review is the taxonomic breadth of species impacted by agricultural change and the consistency of these responses. Agricultural statistics suggest that the rate of change in the structure and management of farmland has slowed in the second half of our review period (e.g. the rate of hedgerow loss has slowed markedly since a peak in the late 1970s). The total amount of pesticide applied has also decreased, but a concurrent increase in efficacy means that there has been little change to its biological impact [30]. There is some suggestion that decreasing rates of environmental change on agricultural land are mirrored in species population trends: the rate of decline in the farmland bird index is lower now than during the 1970s and 80s [31] although this pattern has not been universal.

### Climatic change

Climatic change has had a wide range of impacts on species, with more species impacted positively than negatively in the short-term at least. This pattern has been discussed in detail elsewhere [32,33] and largely reflects the preponderance of species with their northern but not southern range margin in the UK. It may also reflect the bias of studies of climate change impacts towards taxonomic groups where dispersal ability is generally high. Climatic change accounted for a lower proportion of impacts on vascular plants compared to insects and vertebrates, a pattern also found in other reviews [32] and is perhaps not unexpected given the lower dispersal abilities of plants. Many studies of the impact of climatic change investigated the extent of expansion or contraction at the leading or trailing edges of species’ ranges [34,35], therefore giving strong correlative evidence for the impact of climate, whilst not excluding alternative explanations. For instance, Vaughan and Ormerod [36] attributed northwards movement of many freshwater invertebrates to patterns of improvement in water quality, rather than climatic change. In contrast to agricultural change in the UK, surface temperatures are predicted to continue to rise throughout the 21^st^ century under all scenarios of greenhouse gas emissions [37], so we can expect these impacts to increase in coming years [6].

### Habitat change versus habitat management

Our review found that species were, on the whole, impacted more from the management of habitats, than from changes in area covered by them (e.g. intensive agricultural management had a greater impact than increasing farm area). As mentioned previously, wholesale conversion from one habitat type to another was considerable during the middle decades of the 20th century (and earlier). The impacts of habitat conversion may still be felt decades later, through indirect impacts like homogenisation and fragmentation, which were found to be deleterious for many butterfly species in our review. Unlike other types of habitat conversion, urban expansion is accelerating [6]. Urbanisation accounted for a greater impact than other habitat conversion, being a combination of direct conversion of habitat as well as the management of existing urban areas.

### Abandonment of management

A considerable percentage of the absolute impact on species was due to a decline in, or abandonment of, management across farmland, forest, heathland and grassland. Species communities in nearly all UK habitats are adapted to reflect centuries of human management practices, so it is not unexpected that they will be impacted by changes in this management. For instance, a reduction in traditional forest management, and the aging of tree stands [38], resulted in a reduced proportion of forest in early successional stages and was deleterious to a range of species [39] although woodland bats have likely benefitted from the increase in high forest [40]. More recently management has been restored in some cases, often for conservation purposes; early succession species of grassland and heathland have fared better in recent years relative to those occupying similar niches in forests due to recent re-instatement of management in the former two habitats [41].

### Impact of conservation

We show that low-intensity management of agricultural land and habitat creation have had the greatest positive impact on UK species. Agri-environment schemes, whereby farmers are compensated for the cost of applying wildlife-friendly management prescriptions and any associated yield losses, have been implemented throughout the UK. Whilst many agri-environment schemes have been shown to be effective in principle (synopsis in [42]), detecting population level responses has proved difficult [43], but see Bright et al. [44]. Our review indicates that population level impacts are occurring, although their magnitude is still small compared to the negative impacts of intensive management. Of all habitat creation, wetlands have had the biggest impact, with for example birds and Odonata benefitting [45,46]. It appears, however, that the impact of conservation has not been felt equally across taxonomic groups, with vertebrates benefitting the most and vascular plants the least.

### Conclusions and policy implications

Here, we introduce a new structured framework and use it to assess the relative impact of different drivers of change on UK biodiversity in recent decades. The framework allows broad conclusions to be drawn at a range of taxonomic, geographic or temporal scales. It could applied across countries, time periods or for additional taxonomic groups. Our assessment extended to taxonomic groups for which relatively robust evidence was available; we have not tested it outside of the UK’s unusually high level of knowledge on biodiversity and the natural environment.

Our findings have a number of implications for public policy, if the perturbation to, and net loss of, biodiversity in the UK is to be countered: the most important driver of change to address is agriculture, followed by climate change (Figs 2 and 3). A suite of responses are needed to tackle the negative drivers associated with agriculture so that biodiversity can prosper in areas managed for food production. Fundamental changes to public policy, such as the EU Common Agricultural Policy (CAP), are needed both to remove financial support for environmentally harmful production methods, promote sustainable food production, and to support methods that are beneficial for nature. Existing environmental legislation, such as the EU Birds and Habitats Directives, need to be implemented fully and fiscal tools should be explored.

Turning to climate change, the 2015 Paris Climate Conference (COP21) outcomes, particularly the agreed global goal to limit mean surface temperature rises, would limit potential harm to biodiversity. To achieve such ambitious target, countries, including the UK, need to revise emission reduction pledges upwards and to put in place more rigorous mitigation policies. At the same time, we need to promote adaptation policies for the management of land and species, to counter the negative effects and to enhance the positive effects of climate change. As we describe, the net impact of climatic change on UK species in our sample is positive, but it is not clear whether this will always be the case. Protected area networks will be essential to help species survive and track suitable climate space [47]. Well informed site management and creation, along with landscape-scale approaches to improve the quality of land in the matrix between protected sites, will help species to accommodate climate change to come.

Hydrological change and urbanisation come out as the next most important drivers of species decline (Figs 2 and 3). Measures need to be put in place to alleviate and control the negative impacts of water abstraction and drainage on biodiversity. In the existing and planned urban environment, there is a need to increase the area and quality of green space. We also show that different forest practices can impact both negatively and positively on species (Figs 2 and 3); where appropriate, existing woodlands should be brought into sustainable active management to provide rich and varied habitats for specialist forest species, while expanding our native woodland in areas that can benefit nature and people. We should also address the planting decisions of the past with the restoration of heaths, bogs and ancient woodlands whose biodiversity was damaged by inappropriate plantation forestry. Lastly, and on a positive note, habitat creation comes out an important factor driving positive species change (Figs 2 and 3) and this could be enhanced by many different partners from individual land managers and owners, to NGOs, government agencies and businesses. We recommend an accelerated programme of carefully-sited habitat creation projects for priority natural and semi-natural habitats in the UK that would deliver a range of benefits for biodiversity and people.

## Supporting Information

S1 Appendix Results of the drivers of change assessment for all species. (XLSX)

S2 Appendix Results of the drivers of change assessment for each higher taxonomic group. (XLSX)

S3 Appendix Results of the drivers of change assessment for each taxonomic group. (XLSX)

S4 Appendix Species assessments. (XLSX)

S1 Fig Characteristics of the species assessments. Boxplots showing the distribution of a) Strength of Evidence scores, b) Absolute strength of Impact scores (both assessed on a 12 point scale) allocated to each instance of each driver of change listed in the species assessments and c) the number of broad drivers listed per species. For each boxplot the box represents the 25^th^, 50^th^ and 75^th^ percentiles of the distribution and the whiskers represent the maximum and minimum scores and in each case the data are summarised by taxonomic group and shaded by major taxonomic group; vascular plants (green), insects (lemon), vertebrates (coral).
(TIF)

S2 Fig Distribution of Strength of Impacts scores for each broad driver of change. Distribution of Strength of Impact scores for each broad driver of change across all species assessed in the review. The number of instances that the driver was listed in the review is shown in brackets. The distribution of Strength of Evidence scores show a similar pattern.
(TIF)

S3 Fig Relationship between population change and the assessed impact of drivers of change. Relationship between a species’ recent population change (~1970–2012) and the net sum of the impact for all the drivers listed for that species, taking the sign of each impact into account. Only taxonomic groups with comparable population change information (change in abundance or frequency of occurrence) are included. The predicted linear relationship from an ANCOVA is shown for each taxonomic group (ANCOVA results in Table H S1 Text).
(JPEG)

S4 Fig Comparison of different options for weighting species in overall analysis. The percent of absolute impact on species attributable to each Broad driver of change that accounted for two percent of absolute impact or greater, comparing the three options considered for weighting Strength of Impact scores.
(TIF)

S5 Fig Assessment of the influence of low quality evidence in the overall analysis. The percentage of absolute impact on species attributable to each broad driver of change, either using all evidence or using only medium and high quality evidence (Strength of Evidence scores of five or above). Broad drivers accounting for two percent of absolute impact or more are shown.
(TIF)

S6 Fig Results of drivers of change assessment for each taxonomic group. Positive (light green) and Negative (dark blue) impact for each broad driver of change accounting for two percent or more of the total in absolute terms, ordered by absolute impact, by taxonomic group. Impact is shown as a percent of the impact on that group, i.e. absolute impact sums to 100 for each of the three groups. Results are presented using all strength of evidence available.
(TIF)

S1 Text Materials and Methods. (DOCX)
