# Supplementary figures and images for: Agricultural Management and Climatic Change Are the Major Drivers of Biodiversity Change in the UK
Source: PLoS One. 2016 Mar 23;11(3):e0151595. doi: 10.1371/journal.pone.0151595 (PMC4805165; doi:10.1371/journal.pone.0151595)

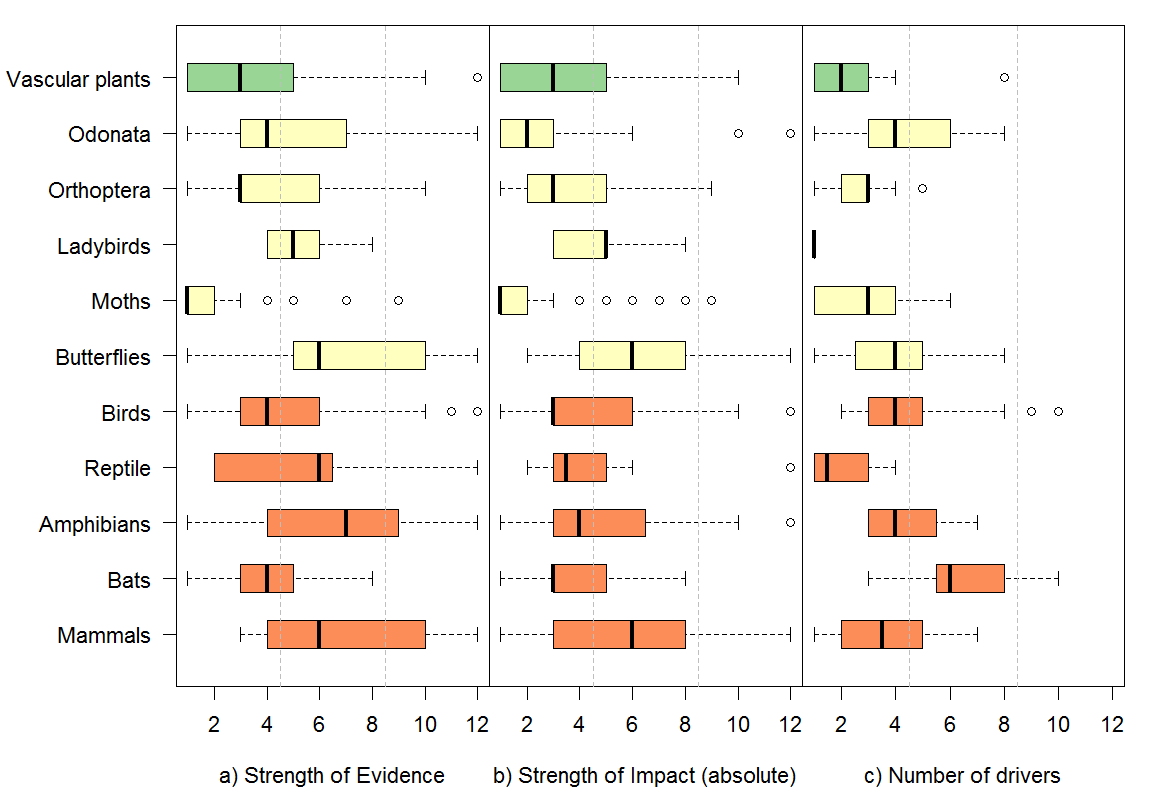

Supplement: S1 Fig — Boxplots showing the distribution of a) Strength of Evidence scores, b) Absolute strength of Impact scores (both assessed on a 12 point scale) allocated to each instance of each driver of change listed in the species assessments and c) the number of broad drivers listed per species. For each boxplot the box represents the 25th, 50th and 75th percentiles of the distribution and the whiskers represent the maximum and minimum scores and in each case the data are summarised by taxonomic group and shaded by major taxonomic group; vascular plants (green), insects (lemon), vertebrates (coral). (TIF) [file pone.0151595.s005.tif]

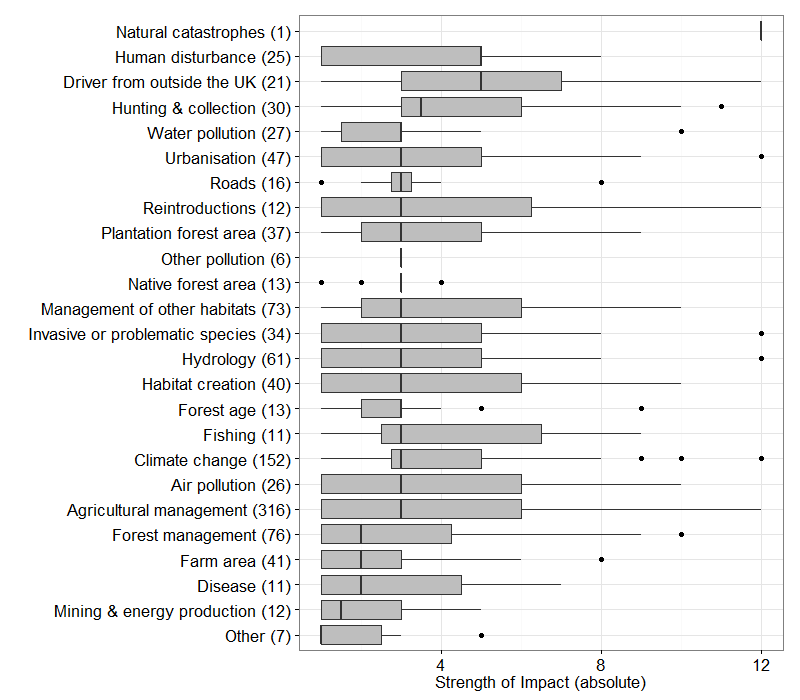

Supplement: S2 Fig — Distribution of Strength of Impact scores for each broad driver of change across all species assessed in the review. The number of instances that the driver was listed in the review is shown in brackets. The distribution of Strength of Evidence scores show a similar pattern. (TIF) [file pone.0151595.s006.tif]

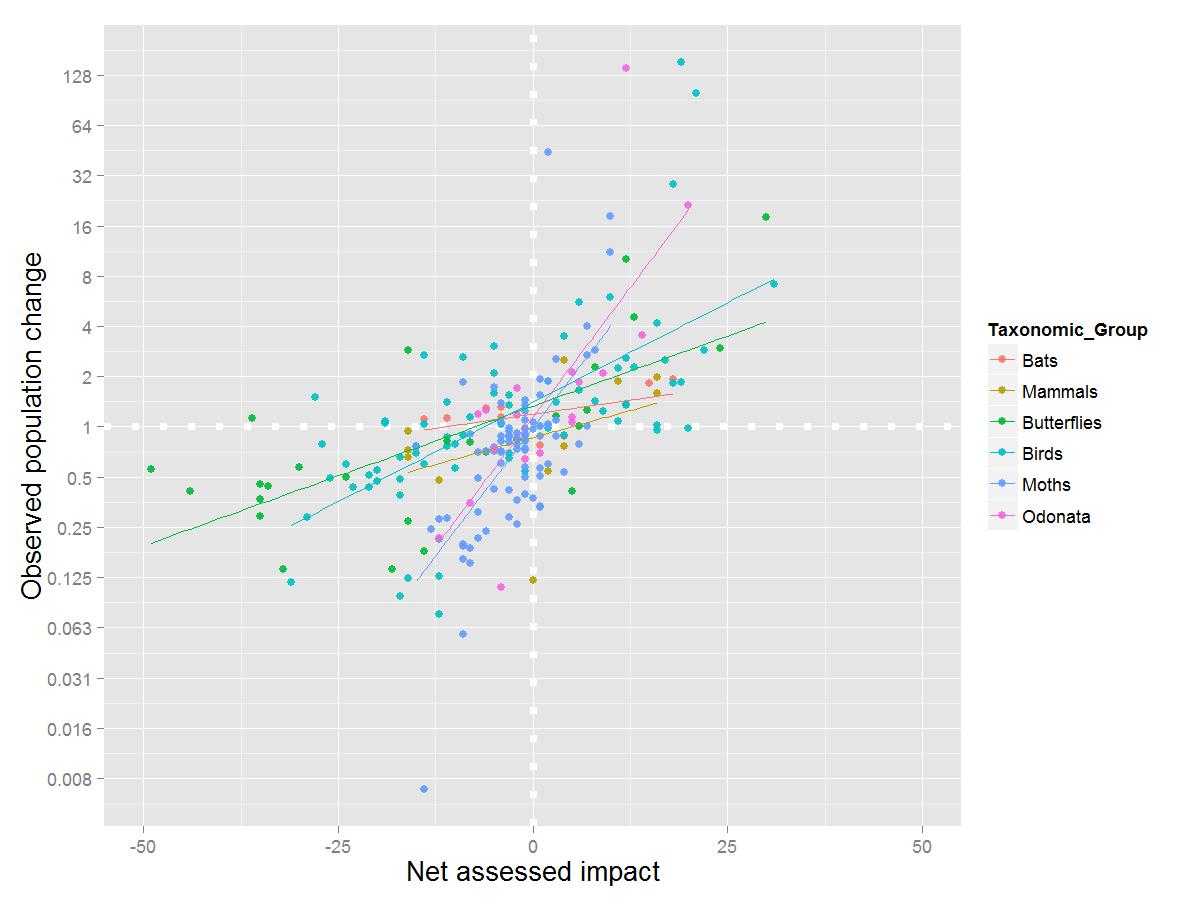

Supplement: S3 Fig — Relationship between a species’ recent population change (~1970–2012) and the net sum of the impact for all the drivers listed for that species, taking the sign of each impact into account. Only taxonomic groups with comparable population change information (change in abundance or frequency of occurrence) are included. The predicted linear relationship from an ANCOVA is shown for each taxonomic group (ANCOVA results in Table H S1 Text). (JPEG) [file pone.0151595.s007.jpeg]

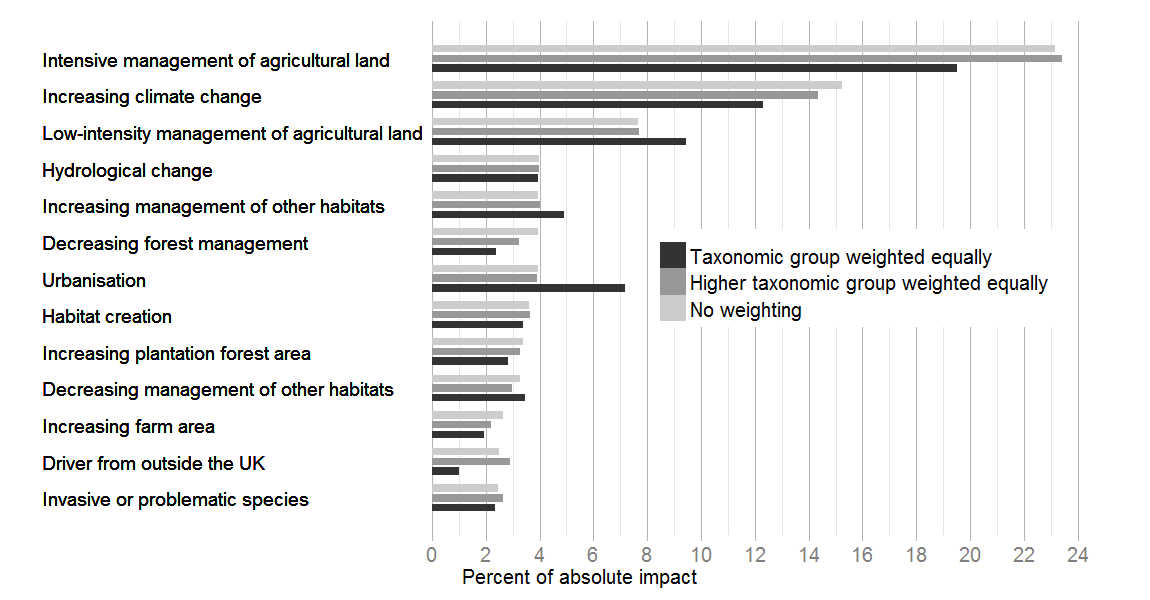

Supplement: S4 Fig — The percent of absolute impact on species attributable to each Broad driver of change that accounted for two percent of absolute impact or greater, comparing the three options considered for weighting Strength of Impact scores. (TIF) [file pone.0151595.s008.tif]

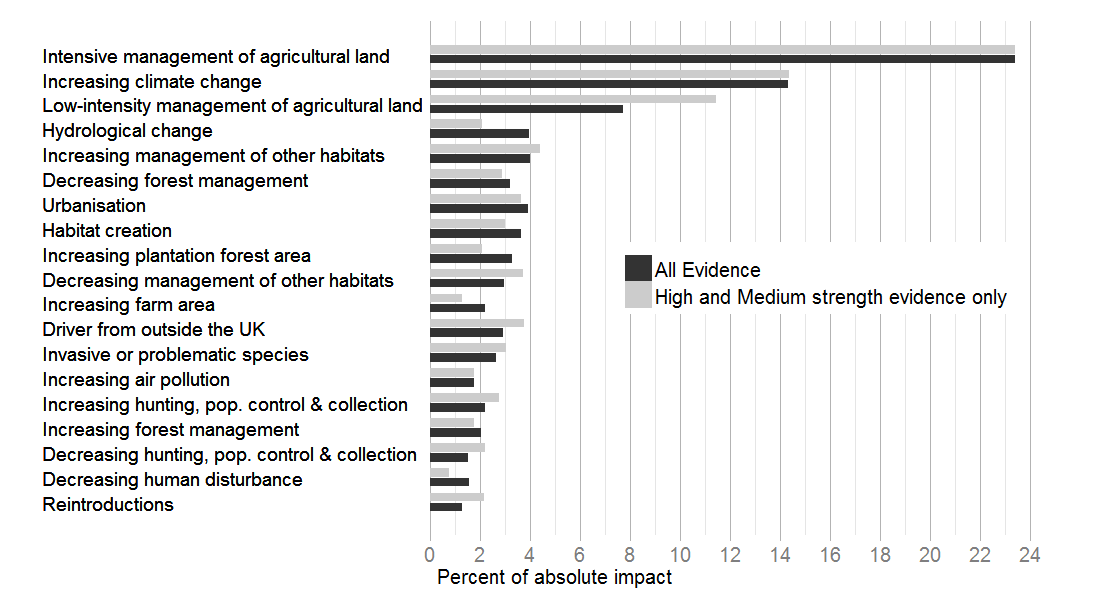

Supplement: S5 Fig — The percentage of absolute impact on species attributable to each broad driver of change, either using all evidence or using only medium and high quality evidence (Strength of Evidence scores of five or above). Broad drivers accounting for two percent of absolute impact or more are shown. (TIF) [file pone.0151595.s009.tif]

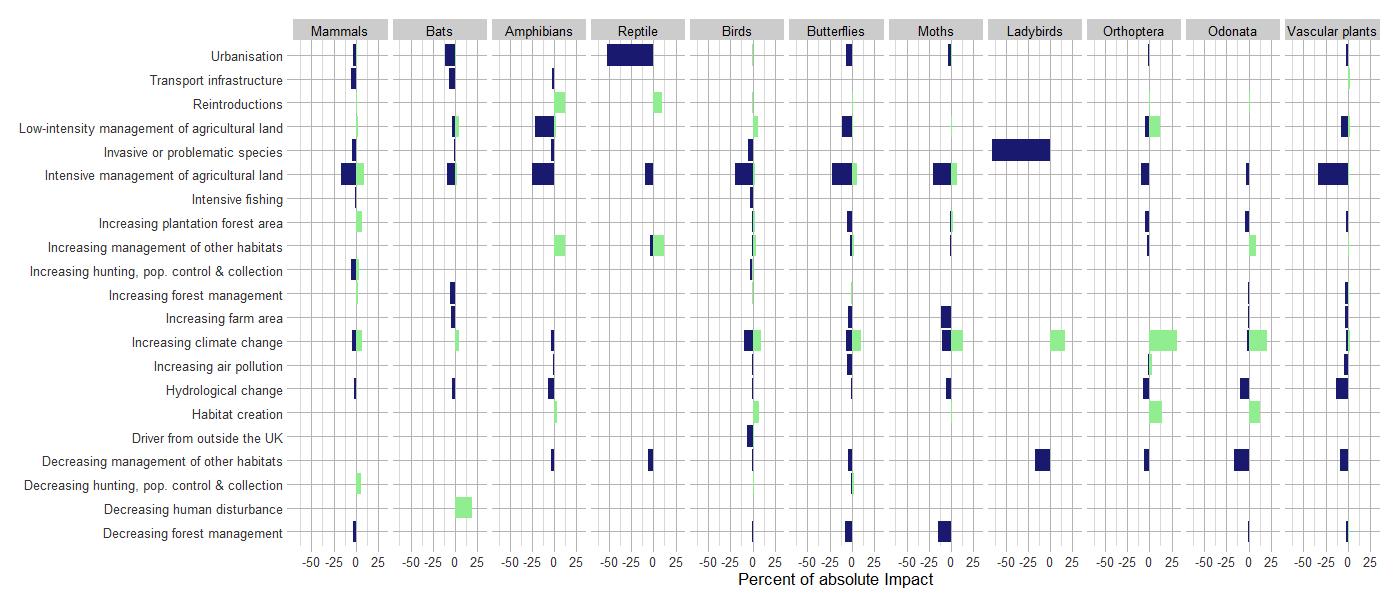

Supplement: S6 Fig — Positive (light green) and Negative (dark blue) impact for each broad driver of change accounting for two percent or more of the total in absolute terms, ordered by absolute impact, by taxonomic group. Impact is shown as a percent of the impact on that group, i.e. absolute impact sums to 100 for each of the three groups. Results are presented using all strength of evidence available. (TIF) [file pone.0151595.s010.tif]
