# Supplementary material for: Agricultural Management and Climatic Change Are the Major Drivers of Biodiversity Change in the UK
Source: PLoS One. 2016 Mar 23;11(3):e0151595. doi: 10.1371/journal.pone.0151595 (PMC4805165; doi:10.1371/journal.pone.0151595)
Supplement: S1 Text — (DOCX) [file pone.0151595.s011.docx]

**S1 Text: Materials and Methods and Results**

**Species selection**

The species in each group with population trends available represent a non-random selection and may not be representative of all species. It might be expected that species for which population trends are available would tend to be more widespread or abundant than average. Where possible, therefore, we compared a measure of rarity to determine whether there was a difference between all species in a taxonomic group and the sub-set with population trends available. In general, that was not the case, although vascular plants with population trends were more widespread than would be expected by chance (Table A). In order to estimate the number of species to sample from speciose groups (birds, butterflies, moths and vascular plants), we repeatedly (1000 times) selected random samples of species of a variety of sample sizes and compared the distribution of population trends, the distribution of species associated with different habitats, and the distribution of species associated with many habitats (generalists), to those associated with few (specialists) between the sample and the full group. Using the simulations as a guide, and through a series of iterations, we took a random sample of species from each taxonomic group and tested whether that differed from the full species list using the same characteristics as described above (Table B). With sample sizes set at 200 for plants, 100 for moths, 30 for butterflies and 70 for birds, we found no significant differences in the characteristics assessed and thus used those sample sizes in our assessment (Table B).

**Metadata and validation**

There was considerable variation in the strength of evidence available to support the species assessments across different taxonomic groups (S1 Fig); with moths, vascular plants and Orthoptera having the lowest median scores. A similar pattern was seen for strength of impact, with moths again having the lowest median. The number of broad drivers of change listed per species was between two and four for the majority of groups, with bats having the greatest number, with a median of six broad drivers listed reflecting species-level knowledge. Scores of strength of evidence and strength of impact were much more consistent across drivers than across taxonomic groups, with a broad range of scores for both parameters listed for most drivers, as well as a low median score (S2 Fig).

In addition to providing clear protocols for each step of the assessment: We used the comments provided by each expert to ensure that the driver categories and the direction of the driver were used consistently, and that the same impact was not recorded more than once under different drivers. If our framework was applied accurately and consistently, we would expect a the net assessed impact experienced by a species (the net sum of the Strength of Impact scores for all drivers listed for the species) to strongly predict the observed species’ population trend. For groups with a comparable measures of population trend (an estimate of the annual rate of change in relative abundance or frequency of occurrence), this relationship was investigated using an ANCOVA, with net impact as a continuous explanatory variable and taxonomic group as a factor. A strong relationship was found (Table H; S3 Fig.; $F_{212}^{1}=126.9;P<0.001$) and the intercept did not significantly differ from zero, suggesting that assessed impact was close to zero for species that have experienced little recent population change. The slope of the relationship differed significantly between taxonomic groups (${F_{212}^{5}F}^{5}=7.80;P<0.001),$ with treatment contrasts showing moths and Odonata to have significantly steeper slopes compared to bats, which have the shallowest (Table H). This means that for a similar observed change in the population the absolute value of assessed impact was less for moths and Odonata compared to bats.

**Weighting Strength of Impact scores and Strength of Evidence scores**

In order to summarise the impact of each driver across all species, we compared three options for weighting the Strength of Impact scores for each species assessment: No weighting (each species is equal), weighting as if we had assessed the same number of species from each higher taxonomic group (Eq1.), and weighting as if we had assessed the same number of species from each taxonomic group (Eq2.). For both equations N = number of species; H = number of higher taxonomic groups; T = number of taxonomic groups; I = Impact scores; and WI = weighted impact score. Subscript letters denote that the parameter is specific to the target higher taxonomic group (h) or taxonomic group (t).

Equation 1: $WI_{h}=I_{h}.\frac{N}{H{.N}_{h}}$ ; Equation 2: $WI_{t}=I_{t}.\frac{N}{T.N_{t}}$

The distributions of impact across broad drivers of change were very similar regardless of whether no weighting was used or strength of impact was weighted as if we had assessed the same number of species from each higher taxonomic group (insects, plants and vertebrates) (S4 Fig; median percentage point difference per driver = 0.11). Full results are given in S1 Appendix. All of the top ten ranked broad drivers were the same, although the order was slightly different. Weighting strength of impact as if we had assessed the same number of species from each taxonomic group led to a slightly different balance of impact between the different drivers of change (median percentage point difference per driver = 0.39). Here eight of the top ten ranked drivers were the same, with some variation in order. We also investigated the impact of reducing the 12 point impact scale to a three point one (low (1), medium (2), high (4)). Results were very similar (S1 and S2 Appendix), and so we chose to retain the 12 point scale at which the assessments had been made.

The taxonomic groups we have included in the review vary considerably in the number of species they include, and in their taxonomic level. For example vascular plants include more than one Phylum, whereas birds are a Class, and ladybirds are a Family. Weighting taxonomic groups equally can elevate the importance of drivers influencing a small number of species only, if they are concentrated in one or more species poor groups. For example, all the top ten broad drivers using the first two options affect 30 or more species each, whereas decreasing human disturbance comes in at position nine when weighting by taxonomic group, and this affects only eleven species, ten of which are bats. Therefore, we chose to use a weight of strength of impact that reflected greater equivalence in number of species assessed within each higher taxonomic group, when presenting our results.

To investigate the influence of low quality evidence on our results, we estimated the Total (absolute), positive and negative impact of each driver of change using all scores of Strength of Evidence and using only medium and high quality evidence; therefore excluding instances where the Strength of Evidence was scored four or less on our scale (Table F). There was a median percentage point difference of 0.25 between the Total (absolute) percent impact assessed using all evidence and that assessed using only high and medium quality evidence (S5 Fig). Therefore, although more than half the impacts (61%) listed in the review were supported by low quality evidence the inclusion of these has limited impact on our overall conclusions. There were a small number of drivers whose impact changed by one percentage point or more when only high and medium quality evidence is used (S1 Appendix). These were low-intensity agriculture, whose impact was elevated when using high and medium quality evidence alone meaning that it is supported by higher than average quality evidence, whereas the impact of hydrological change and increasing area of plantation forest were reduced. This impact, albeit limited, reduces the representativeness of our review and therefore, we include all qualities of evidence in our results.

**Table A: Characteristics of the taxonomic groups included in the assessment of drivers**

| Group | Taxa, Sampled [with population trends available; in group] | Population change measure | Monitoring/ recording scheme(s) | Are species with population trends available more or less rare than expected? [Measure (Statistic)]^a^ |
| --- | --- | --- | --- | --- |
| Vertebrates | Mammals (not bats), 14 [14; 29] | Abundance* | BBS^1^, NDMP^2^, Otter surveys^3^, Badger survey^4^ | NA |
|  | Bats, 11 [11; 18] | Abundance | NBMP^5^ | NA |
|  | Birds, 70 [198; 250] | Abundance | BBS, WeBS^6^, SMP^7^, RBBP^8^, SCARRABS | Population size ($Z=-0.556;P=0.57$) |
|  | Amphibians, 7 [7; 7] | Abundance (Qualitative) | Questionnaire^9^ | NA |
|  | Reptiles, 6 [6; 6] | Abundance (Qualitative) | Questionnaire^10^ | NA |
| Invertebrates | Butterflies, 30 [56; 59] | Abundance | UKBMS ^11^ | NA |
|  | Ladybirds, 25[25; 47] | Frequency of Occurrence | UK Ladybird survey^12^ | ISIS rarity score ($Z=0.405;P=0.686$) |
|  | Orthoptera, 11 [11; 30] | Distribution | Orthoptera recording scheme^13^ | ISIS rarity score ($Z=0.0108;P=0.991$) |
|  | Odonata, 20 [39; 52] | Frequency of Occurrence | British Dragonfly Society, Dragonfly Recording Network ^14^ | ISIS rarity score ( $Z=-0.00697;P=0.994$) |
|  | Moths, 95 [648; 882] | Abundance/ Frequency of Occurrence | Rothamsted Insect Survey^15^, NMRS^16^ | ISIS rarity score ($X_{1}^{2}=0.0024; P = 0.961$ |
| Plants | Vascular plants, 109 [1309; 1546] | Distribution | Plant Atlas^17^ | UK Range size ($Z=-3.254;P=0.001***$) |
| **Total** | **398** |  |  |  |

* For otter and water vole change in distribution measured

a: Population size or rarity scores for species with available population trends were compared to those for the entire taxonomic group using a z-test, or chi-squared test.

**Table B: Comparison of the characteristics of the species sampled within each taxa to all species within the taxa with population trends available**

| Attribute | Taxonomic Group | | | |
| --- | --- | --- | --- | --- |
|  | Plants (200) | Moths (100) | Butterflies (30) | Birds (70) |
| Population change (1970-2010) | $Z=0.731;P=0.435$ | $Z=0.534;P= 0.594$ | $X_{1}^{2}=0.286;P=0.593$ | $Z=0.489;P= 0.625$ |
| Distribution of species amongst State of Nature broad habitats | $X_{6}^{2}=2.465;P=0.872$ | $X_{6}^{2}=0.954;P=0.987$ | $X_{6}^{2}=2.877;P=0.824$ | $X_{1}^{4}=1.0286;P=0.905$ |
| No. of habitats each species is associated with (a measure of breadth of ecological niche) | $X_{2}^{2}=0.884;P=0.643$ | $X_{7}^{2}=4.30;P=0.744$ | $X_{4}^{2}=0.745;P=0.946$ | NA |

**Table C: Broad and specific driver of change categories, with direction of driver specified as high or low anthropogenic impact and conservation action or not.**

| Broad driver | Direction of Broad driver | Direction of specific driver | Explanation | Conservation action | Anthropogenic impact | Link to IUCN\|EU Directives drivers |
| --- | --- | --- | --- | --- | --- | --- |
| Agricultural management | Intensive management of agricultural land | production driven farm practices | Timing – sowing, mowing, Crop choice, Rotation, mixed farming |  | High | A02, A05, A06\| 2.1, 2. |
|  |  | intensive grazing regime |  |  | High |  |
|  |  | loss of semi-natural habitat | Frequency of semi-natural habitat, e.g. ponds, hedgerows |  | High |  |
|  |  | re-instatement of management | Including a restoration of grazing | Y | High |  |
|  |  | increasing fertiliser use | Terrestrial impact, use water pollution for freshwater |  | High | A08\| 9.3.3 |
|  |  | increasing pesticide, herbicide use |  |  | High | A07\| 5.3 |
|  |  | General | General intensification of agriculture |  | High | A07\| 5.4 |
|  | Low-intensity management of agricultural land | sustainable farm practices | Timing – sowing, mowing, Crop choice, Rotation, mixed farming | Y | Low | A02, A05, A06\| 2.1, 2.3 |
|  |  | moderate grazing regime |  | Y | Low |  |
|  |  | increasing semi-natural habitat | Frequency of semi-natural habitat, e.g. ponds, hedgerows | Y | Low |  |
|  |  | lack of management | Including a move towards undergrazing |  | Low |  |
|  |  | reduced fertiliser use | Terrestrial impact, use water pollution for freshwater | Y | Low | A08\| 9.3.3 |
|  |  | reduced pesticide, herbicide use |  | Y | Low | A07\| 5.3 |
|  |  | General | General intensification of agriculture | Y | Low | A07\| 5.4 |
| Air pollution | Increasing air pollution | nitrogen |  |  | High | H04.02\|9.5 |
|  |  | sulphur dioxide |  |  | High |  |
|  |  | Other |  |  | High | H04\|9.1, 9.2 |
|  |  | General | All types of air pollution |  | High | NA |
|  | Decreasing air pollution | nitrogen |  | Y | Low | H04.02\|9.5  H04.01\|9.5 |
|  |  | sulphur dioxide |  | Y | Low |  |
|  |  | Other |  | Y | Low | H04\|9.1, 9.2 |
|  |  | General | All types of air pollution | Y | Low | NA |
| Climate change | Mitigation of climate change | mitigation of sea level rise |  | Y | Low | M01.07\|11 |
|  |  | mitigation of changing climatic conditions | Change to temperature, rainfall | Y | Low | M01.(01-06) |
|  | Increasing climate change | sea level rise | habitat loss, coastal squeeze |  | High | M01.07\|11 |
|  |  | changing climatic conditions | Change to temperature, rainfall |  | High | M01.(01-06) |
| Disease | Reduction in disease |  |  |  | Low | 7.2 |
|  | Disease |  |  |  | High |  |
| Farm area | Increasing farm area |  |  |  | High | A01\|2.1, 2.3 |
| Fishing | Sustainable fishing |  | includes effects of by-catch/accidental catch | Y | Low | F02\|5.1.1, 5.2 |
|  | Intensive fishing |  |  |  | High |  |
| Forest management | Increasing forest management | traditional forest management | Coppicing, pollarding, selective felling. | Y | High | B02\|2.2, 5.3 |
|  |  | loss of important habitat features | Veteran trees, deadwood |  | High |  |
|  |  | intensive grazing regime |  |  | High | B02\|2.2, 5.3 |
|  | Decreasing forest management | lack of traditional forest management | Coppicing, pollarding, selective felling |  | Low |  |
|  |  | important habitat features available | Veteran trees, deadwood | Y | Low |  |
|  |  | moderate grazing regime |  | Y | Low |  |
| Habitat creation | Habitat creation | Wetland | Implies loss of other habitat, link to which | Y | Low |  |
|  |  | Coastal |  | Y | Low |  |
|  |  | Upland |  | Y | Low |  |
|  |  | Heathland |  | Y | Low |  |
|  |  | semi-natural grassland |  | Y | Low |  |
| Habitat management-other | Increasing management of other habitats | General | General increase or decrease in management | Y | High |  |
|  |  | intensive grazing regime |  |  | High | 7.1 |
|  |  | controlled burning |  |  | High |  |
|  |  | use of habitat products or habitat | Thatch, bracken etc. | Y | High |  |
|  |  | stabilisation of ephemeral habitat | Dune slacks, sand dunes etc. |  | High |  |
|  |  | re-instatement of management | Undergrazing | Y | High |  |
|  | Decreasing management of other habitats | General | General increase or decrease in management |  | Low |  |
|  |  | moderate grazing regime |  | Y | Low | 7.1 |
|  |  | reduction in controlled burning |  |  | Low |  |
|  |  | lack of use of habitat products or habitat | Thatch, bracken etc. |  | Low |  |
|  |  | ephemeral habitat maintained | Dune slacks, sand dunes etc. | Y | Low |  |
|  |  | lack of management | Undergrazing |  | Low |  |
| Human disturbance | Increasing human disturbance |  |  |  | High | G\|9.5 |
|  | Decreasing human disturbance |  |  | Y | Low | G\|9.5 |
| Hunting & collection | Increasing hunting, pop. control & collection | unsustainable hunting and collection |  |  | High | F03.01, F03.02.01\| 5.1.2, 5.1.3 |
|  |  | population control | E.g. Foxes, corvids, rabbits |  | High | F03.02 |
|  |  | widespread stocking | Game birds |  | High | F03.01.01\|6 |
|  | Decreasing hunting, pop. control & collection | sustainable hunting and collection |  | Y | Low | F03.01, F03.02.01\| 5.1.2, 5.1.3 |
|  |  | reduced population control | E.g. Foxes, corvids, rabbits |  | Low | F03.02 |
|  |  | reduced stocking | Game birds | Y | Low | F03.01.01\|6 |
| Hydrology | Hydrological change | increasing abstraction and drainage |  |  | High | J02.(01-05,10-12)\|7.3 |
|  |  | increasing physical modification of freshwater habitats | Alteration of course, canalisation, riparian management |  | High | J02.(01-05,10-12)\|7.3 |
|  | Alleviation of hydrological change | reduction in abstraction and drainage |  | Y | Low | J02.(01-05,10-12)\|7.3 |
|  |  | mitigation of physical modification of freshwater habitats | Alteration of course, canalisation, riparian management | Y | Low |  |
| Invasive or problematic species | Invasive or problematic species |  |  |  | High | I |
|  | Alleviating invasive species |  |  | Y | Low | I |
| Mining & energy production | Increasing mining & energy production |  | Includes Renewables |  | High | C\| 4  C\| 4 |
|  | Decreasing mining & energy production |  |  | Y | Low |  |
| natural catastrophes | Reduction in natural catastrophes |  |  |  |  | L\|11  L\|11 |
|  | Increase in natural catastrophes |  |  |  |  |  |
| No drivers of change |  |  |  |  |  | X:12 |
| No known driver |  |  |  |  |  |  |
| Driver from outside the UK | Driver from outside the UK |  | Specify if known, choosing from options above |  | High |  |
| Other |  |  |  |  |  | 2.1, 2.3 |
| other pollution | Increasing other pollution |  | e.g. Noise, light pollution |  | High | H\|8 |
|  | Decreasing other pollution |  | e.g. Noise, light pollution | Y | Low |  |
| Plantation forest area | Increasing plantation forest area |  | Includes native forests planted in inappropriate areas |  | High | B01, B03\|2.2, 5.3 |
| Reintroductions | Reintroductions |  |  | Y | High |  |
| Roads | Transport infrastructure |  |  |  | High | D\|1 |
|  | Mitigation of transport infrastructure |  |  | Y | Low | D\|1 |
| Unknown |  |  |  |  | NA |  |
| Urbanisation | Urbanisation |  | Includes changes in construction and restoration methods |  | High | E\|2.4, 5.4 |
|  | Mitigation of urbanisation |  |  | Y | Low |  |
| Water pollution | Increasing water pollution | nitrogen |  |  | High | H04.02\|9.5 |
|  |  | other |  |  | High | H04\|9.1, 9.2 |
|  | Decreasing water pollution | nitrogen |  | Y | Low | H04.02\|9.5 |
|  |  | other |  | Y | Low | H04\|9.1, 9.2 |
| Forest age | Increasing forest age | NA |  |  | Low | 3 |
|  | Decreasing forest age | NA |  |  | High | 3 |
| Native forest area | Increasing native forest area | NA | Assumes increases are in appropriate areas | Y | Low | B03\| 2.2, 5.3 |

**Table D: Taxonomic experts contributing to species assessment process**

| Taxa | Experts |
| --- | --- |
| Birds | Ian Henderson, Mark Eaton, Fiona Burns, Jennifer Smart, Paul Bellamy, Tony Morris, Staffan Roos, Steven Ewing, David Douglas, Simon Wotton, Andy Stanbury |
| Mammals | Nida al Fulaij, Dave Williams, Richard Yarnell, Dawn Scott, David Wembridge, Graham Scholey, Jenny MacPherson, Tom Moorhouse, Johnny Birks, Neil Reid, Pat Morris, Silviu Petrovan, Dave Mallon, Lucy Lush |
| Bats | Kate Barlow, Karen Haysom, Carol Williams |
| Amphibians | Fiona Burns, John Wilkinson, Tony Gent, Jim Foster |
| Reptiles | Fiona Burns, John Wilkinson, Tony Gent, Jim Foster |
| Butterflies | Tom Brereton, Martin Warren, Caroline Bulman, Ian Middlebrook, Sam Ellis |
| Moths | David Brooks, Mark Parsons |
| Odonata | Fiona Burns, Dave Smallshire, Claire Install, Chris Meredith, Stephen Prentice, the BDS Conservation Group. |
| Orthoptera | Bjorn Beckmann, Tim Gardiner, Ted Benton |
| Ladybirds | Helen Roy, Peter Brown |
| Plants | Peter Stroh, Kevin Walker, Fiona Burns, David Pearman, Owen Mountford, Simon Smart |

**Table E: Definitions used to score the strength of Impact of a driver on a species**

| Score | Category | Definition |
| --- | --- | --- |
| 12 | Major positive/ negative impact | Explain all or the majority of a very large population change |
| 11 |  |  |
| 10 |  | Explaining a major part of a very large population change or all of a substantial change |
| 9 |  |  |
| 8 | Moderate positive/ negative impact | Explaining a major part of a substantial population change or a moderate change |
| 7 |  |  |
| 6 |  |  |
| 5 |  | Explaining a moderate part of a substantial population change or a small/ moderate change |
| 4 | Low positive/ negative impact |  |
| 3 |  | Contributing factor to population change or explains a substantial part of a small change |
| 2 |  |  |
| 1 |  | Explaining local impact on population, has only marginal impact on national population |
| ? |  | If it is not possible to score strength of impact |

**Table F: Definitions used to score the strength of evidence for each driver**

| Score | Category | Definition | Response variables |
| --- | --- | --- | --- |
| 12 | High | Strong robust evidence showing the presence and strength of the relationship between a driver and species change. Likely to be peer reviewed papers with either a observational or experimental approach, at a broad spatial and temporal scale with no substantial biases known and alternative explanatory variables controlled for. | Population size, density, frequency of occurrence, presence, adult survival |
| 11 |  |  |  |
| 10 |  | Good evidence (some deviations from ideal described above) for presence of and some indication of strength of relationship between a driver and species change. |  |
| 9 |  |  |  |
| 8 | Medium | Reasonable quality and quantity of evidence for presence of a relationship between a driver and species change | All of the above, plus productivity measures, juvenile survival and habitat selection |
| 7 |  |  |  |
| 6 |  |  |  |
| 5 |  | Some quantitative evidence for relationship between driver and species change, studies may be of small scale either temporally or spatially. |  |
| 4 | Low |  | Any of the above |
| 3 |  | Can explain rationale for believing there to be a relationship between driver and species change, for example personal observations. |  |
| 2 |  |  |  |
| 1 |  | Relationship postulated between driver and species change |  |
| Indirect evidence: Evidence for one species may be extrapolated to similar species where it can be argued that they are likely to be sensitive to the same environmental parameters. If indirect evidence of this type is used then it should be downgraded by one category of data quality. | | | |
| Non-UK evidence: People are encouraged to use evidence from outwith the UK. Judgement must be used to assess how strong this evidence is depending on how similar the species’ situation is where the study was completed to its situation in the UK. | | | |

**Table G: Published information on the average change in environmental drivers, collated to advise the review**

| **Driver of Change** | **Data** | **Scale** | **1970 (or nearest - to 1975)** | **1980 (or nearest - 1976 - 1985)** | **1990 (or nearest 1986 - 1995)** | **2000 (or nearest 1996 - 2005)** | **2010 (or nearest 2006 - 2013)** |
| --- | --- | --- | --- | --- | --- | --- | --- |
| **Agricultural area** | BAP Broad Habitat Area (000 ha) Arable & Horticulture^18^ | GB |  | 5283.0 | 5024.6 | 5067.4 | 4608.5 |
|  | BAP Broad Habitat Area (000 ha) Boundary & linear features^18^ | GB |  |  | 580.7 | 511.3 | 496.1 |
|  | BAP Broad Habitat Area (000 ha) Improved Grass^18^ | GB |  | 5903.0 | 4619.0 | 4251.2 | 4493.6 |
|  | BAP Broad Habitat Area (000 ha) Neutral Grass^18^ | GB |  | 467.0 | 1669.5 | 2006.8 | 2176.3 |
|  | BAP Broad Habitat Area (000 ha) Ca Grass^18^ | GB |  | 75.0 | 78.2 | 61.5 | 57.0 |
|  | BAP Broad Habitat Area (000 ha) Acid Grass^18^ | GB |  | 1476.0 | 1821.4 | 1502.5 | 1588.8 |
|  | Extent of semi-natural grassland^19^ | E/W | Huge decrease in unimproved pasture and rough grazing 1930-1984; a steep decline in the 1940's and steady decline otherwise throughout the period (Fuller 1987) | | | | |
| **Agricultural management** | Higher level Agri-Environment Schemes (Ma ha)^20^ | UK |  |  | 0.308 | 2.077 | 2.899 |
|  | Entry level Agri-Environment Schemes (Ma Ha)^20^ | GB |  |  |  | 0.031 | 6.236 |
|  | BAP Priority Habitat – Hedges^18^ | GB |  | 624 | 506 | 508 | 477 |
|  | Hedges: av. annual chg (‘000km/year)^21^ | E | -3.9 | -5.5 |  |  |  |
|  | Wheat Yield Structure of the agriculture industry^22^ | UK | 4.19 | 5.88 | 6.97 | 8.01 | 7.7 |
|  | Permanent grass (excl rough grazing) (000 ha) June Census of Agriculture: 1900-2010 | E | 3 261.5 | 3 154.9 | 3 106.9 | 2 876.6 | 3 288.4 |
|  | Overall nitrogen application rates (kg/ha)^23^ | E/ W | 89 | 120 | 141 | 124 | 105 |
|  | Overall phosphate application rates (kg/ha)^23^ | E/ W | 39 | 37 | 38 | 31 | 18 |
|  | Overall potash application rates (kg/ha) | E/ W | 36 | 40 | 49 | 39 | 23 |
|  | Area of major crops treated with herbicide as a % of crop area, can be >100% as more than one pesticide often used each year^24^ | E |  |  | 225.1 | 310.3 | 434.8 |
|  | Area of major crops treated with insecticide as a % of crop, can be >100% as more than one pesticide often used each year^24^ | E |  |  | 62.3 | 92.0 | 132.9 |
|  | Pesticide use on cereals kg a.s./ha crop grown^24^ | E |  |  | 4.7 | 3.9 | 3.31 |
|  | Total Cattle (thousand head at June)^22^ | UK | 12 581.0 | 13 426.0 | 12 192.4 | 11 134.6 | 10 111.7 |
|  | Total sheep and lambs (thousand head at June) | UK | 26 100.0 | 31 400.0 | 44 469.4 | 42 264.1 | 31 084.3 |
|  | Average yield per dairy cow (L annum^-1^) | UK | 3975.0 | 4727.0 | 5151.0 | 5978.6 | 7272.9 |
| **Forest area (Native)** | BAP Broad Habitat Area (000 ha) Broadleaved, mixed & Yew Woodland^18^ | GB |  | 1317.0 | 1343.2 | 1328.3 | 1406.0 |
| **Forest area (Plantation - native or introduced)** | BAP Broad Habitat Area (000 ha) Coniferous woodland^18^ | GB |  | 1243.0 | 1239.0 | 1385.9 | 1318.7 |
| **Forest area** | Total Woodland Area^19^ | UK | **total** woodland cover 7.4% 1965 & 11.7 in 2009 | | | | |
| **Forest management** | Percentage sustainably managed forest^20^ | UK |  |  |  | 35.710 | 43.201 |
|  | Ratio of coppice/scrub to high forest^25^ | E | 1947: 49% of broadleaved woodland coppice or scrub & 51% high forest. 2002: high forest - 97% | | | | |
|  | Red deer pop, Gamebag census, % change from yr to 2010^26^ | UK | 174* | 40* | -1.0 |  |  |
|  | Red deer pop, BBS % change from yr to 2010^1^ | UK |  |  | 99* |  |  |
|  | Sika deer pop, Gamebag census % change from yr to 2010^26^ | UK |  | 161* | 61* |  |  |
|  | Sika deer pop, BBS % change from yr to 2010^1^ | UK |  |  |  |  |  |
|  | Fallow deer pop, Gamebag census % change from yr to 2010^26^ | UK | 370.0 | 84 | 30 |  |  |
|  | Fallow deer pop, BBS, % change from yr to 2010^1^ | UK |  |  | 35.0 |  |  |
|  | Roe deer pop, Gamebag census % change from yr to 2010^26^ | UK | 660* | 104* | 49* |  |  |
|  | Roe deer pop, BBS % change from yr to 2010^1^ | UK |  |  | 58* |  |  |
|  | Muntjac pop, Gamebag census % change from yr to 2010^26^ | UK | 1756* | 1148* | 210* |  |  |
|  | Muntjac pop, BBS, % change from yr to 2010^1^ | UK |  |  | 67* |  |  |
|  | Grey squirrel pop, Gamebag census % change from yr to 2010^26^ | UK | 97* | 113* | 59* |  |  |
|  | Grey squirrel pop, BBS % change from yr to 2010^1^ | UK |  |  | 53* |  |  |
| **Mining & energy production** | Renewable Energy Capacity (MW)^27^ | UK |  |  |  |  | 9215.0 |
|  | Electricity generation^27^ | UK | Renewable share of electricity generation ~7% 2010,~12% 2013 | | | | |
|  | Sales of 1^o^ aggregates - sand & gravel^28^ | E/ W | 119,384 | 89,324 | 119,238 | 80,004 | 48,317 |
| **Transportation** | Road length (km)^29^ | UK | 322484 | 339633 | 358034.3 | 390237 | 394253.1 |
|  | Traffic Volume (vehicle km)^30^ | UK | 200.5 | 271.9 | 410.8 | 466.2 | 487.9 |
| **Urbanisation & development** | BAP Broad Habitat Area (000 ha) - Built-up areas & gardens^18^ | GB |  | 1266.0 | 1265.5 | 1279.4 | 1323.4 |
| **Fishing, aquatic resources** | Proportion of large fish (=> 40cm), by weight, in the N North Sea^20^ | UK |  | 23.33 | 9.97 | 5.01 | 8.84 |
|  | Fish stocks harvested sustainably & at full reproductive capacity^20^ | UK |  |  | 28.6 | 26.7 | 40.0 |
| **Pollution, air** | % area of sensitive habitats > acidity critical loads^20^ | UK |  |  |  | 60.3 | 48.7 |
|  | % area of sensitive habitats > nutrient N critical loads^20^ | UK |  |  |  | 68.7 | 67.5 |
|  | Nitrogen Oxides Emissions (Ma T)^31^ | UK | 2.6 | 2.7 | 2.9 | 1.8 | 1.1 |
|  | Sulphur Dioxide Emissions (Ma T) ^31^ | UK | 6.4 | 4.8 | 3.7 | 1.2 | 0.4 |
|  | Nitrogen Deposition^32^ | UK | The total deposition of N in the UK changed little 1987 - 2006 | | | | |
|  | Sulphur Deposition^32^ | UK | Concentrations of sulphur dioxide in UK surface air have declined to values which no longer pose a direct threat to sensitive plant species. Sulphur deposited in the UK has decreased by 80% 1986-2006. There has been a reduction in the area of Broad Habitats exceeding critical loads for acidity from 71% in 1996-98 to 54% in 2006-08. | | | | |
|  | Ozone^32^ | UK | Peak ground level ozone (O3) concentrations declined by about 30% 1986-2007, while annual mean ozone concentrations in the UK increased by 0.2 ppb per year, representing an increase of approximately 15% over the 20 years. Critical levels of ozone for agricultural crops, forests and semi-rural vegetation are exceeded over much of the UK. | | | | |
| **Pollution, water** | % of total river length of 'good' biological quantity^20^ | E |  |  | 62.6 | 69.1 | 72.5 |
|  |  | W |  |  | 79.7 | 77.6 | 87.1 |
|  | % of total river length of 'good' biological quantity - new system^20^ | NI |  |  |  | 61.5 | 53.8 |
|  |  | S |  |  |  | 86.7 | 87.6 |
|  | % of total river length of 'good' biological quantity - old system^20^ | NI |  |  | 76.1 | 66.1 |  |
| **Pollution, other** | Input of hazardous substances to the marine environment^20^ | UK |  |  | 100.0 | 61.5 | 20.9 |
| **Invasive species** | No. of Invasive species present in 10% or more of potential range^20^ | GB | 6 | 10 | 16 | 19 | 24 |
| **Hydrology** | BAP Broad Habitat Area (000 ha) Standing Open Water & Canals^18^ | GB |  | 284 | 200 | 196 | 204 |
|  | BAP Broad Habitat Area (000 ha) Rivers & Streams^18^ | GB |  | 70.0 | 70.0 | 65.0 | 58.0 |
|  | BAP Priority Habitat Ponds^18^ | GB |  |  |  | 425.0 | 478.0 |
| **Climate change** | Sea level change^19^ | UK | ~1mm per year across the 20th century | | | | |
|  | Surface Temperature^33^ | E | During the 20th century, the annual mean central England temperature increased by about 0.9 °C. The last decade was exceptionally warm in central England by historical standards, on average about 0.78 °C warmer than the 1961-1990 average. | | | | |
|  | Precipitation^34^ | UK | Rainfall in the UK is highly variable from month to month and year to year, making it difficult to detect or attribute the cause of long-term trends. For the UK as a whole, recent decades have been getting wetter but this does mask a lot of regional and seasonal differences and may be part of temporal variability. There are also signs that a greater proportion of rain is falling in more intense events. | | | | |
|  | Extreme weather events^34^ | UK | In the last decade or so, a number of extreme months or seasons have occurred in the weather record. In some cases, such as the European heat wave of summer 2003, there is evidence that human influence has changed the odds of such warm events happening. | | | | |
| **Habitat management/ restoration (other than Ag/Forests)** | Extent of nationally and internationally important protected areas (Ma ha) ^20^ | UK | 2.81 | 4.24 | 5.09 | 5.62 | 6.68 |
|  | Prop of ASSI (NI), SSSI (E, W, S) in favourable condition^20^ | UK |  |  |  | 53.70 | 52.93 |
|  | Proportion of ASSI (NI), SSSI (E, W, S) in unfavourable recovering condition^20^ | UK |  |  |  | 13.65 | 30.91 |
|  | Rabbit pop, Gamebag census % change from yr to 2010^26^ | UK | 1046* | 109* | -24 |  |  |
|  | Rabbit pop, BBS % change from that yr to 2010^1^ | UK |  |  | -46* |  |  |
| **Habitat recreation (other than Forests)** | BAP Broad Habitat Area (000 ha) Bracken^18^ | GB |  | 439 | 271.64 | 314.57 | 260.07 |
|  | BAP Broad Habitat Area (000 ha) Dwarf Shrub Heath^18^ | GB |  | 1388.0 | 1436.3 | 1299.1 | 1342.7 |
|  | BAP Broad Habitat Area (000 ha) Fen, Marsh & Swamp^18^ | GB |  | 428.0 | 426.8 | 425.5 | 391.9 |
|  | BAP Broad Habitat Area (000 ha) Bog^18^ | GB |  | 2303.0 | 2050.0 | 2222.1 | 2231.8 |
|  | BAP Priority Habitat Lowland Dwarf Shrub Heath^18^ | GB |  |  |  | 78.0 | 93.0 |
|  |  | E |  |  |  | 27.0 | 44.0 |

**Table H: Results of an ANCOVA testing whether the net assessed impact for each species in our review predicts the observed recent population change and whether this relationship varies between taxonomic groups. Treatment contrasts are presented.**

| Parameter | Estimate | s.e. | t | P |
| --- | --- | --- | --- | --- |
| Intercept | 0.176 | 0.283 | 0.620 | 0.536 |
| Net impact | 0.0155 | 0.0290 | 0.534 | 0.594 |
| Mammals | -0.323 | 0.384 | -0.841 | 0.401 |
| Butterflies | 0.113 | 0.347 | 0.325 | 0.745 |
| Birds | 0.165 | 0.303 | 0.543 | 0.588 |
| Moths | -0.191 | 0.302 | -0.634 | 0.527 |
| Odonata | -0.0414 | 0.356 | -0.116 | 0.907 |
| Net impact : Mammals | 0.0143 | 0.0367 | 0.388 | 0.698 |
| Net impact: Butterflies | 0.0231 | 0.0302 | 0.765 | 0.445 |
| Net impact: Birds | 0.0393 | 0.02986 | 1.317 | 0.189 |
| Net impact: Moths | 0.126 | 0.0348 | 3.606 | **<0.001** |
| Net impact: Odonata | 0.128 | 0.0387 | 3.299 | **0.001** |
| $F_{212}^{11}=17;P=<0.001;adjR^{2}=0.441$ | | | | |

**References**

1. Risely, K., et al., The Breeding Bird Survey 2012, BTO Research Report 645. 2013, British Trust for Ornithology: Thetford.

2. PTES, National Dormouse Monitoring Programme. 2012, People's Trust for Endangered Species: London.

3. EA, Fifth otter survey of England 2009 - 2010 Summary report in Environment Agency. 2010, Environment Agency: Bristol.

4. Judge, J., Wilson, G.J., Macarthur, R., Delahay, R.J., McDonald, R.A., 2014. Density and abundance of badger social groups in England and Wales in 2011–2013. Scientific reports 4.

5. Barlow, K., Briggs, P., Haysom, K., Hutson, A., Lechiara, N., Racey, P., Walsh, A., Langton, S., 2015. Citizen science reveals trends in bat populations: The national bat monitoring programme in Great Britain. Biological Conservation 182, 14-26.

6. Holt, C., et al., Waterbirds in the UK 2010/11: The Wetland Bird Survey. 2012, BTO: Thetford.

7. JNCC. 2012. Seabird population trends and causes of change. JNCC, Peterborough..

8. Holling, M., Rare breeding birds in the United Kingdom in 2008. British Birds, 2010. **103**: p. 482-538.

9. Cooke, A.S. and H.R.A. Scorgie, The Status of the Commoner Amphibians and Reptiles in Britain, Focus on Nature Conservation 3. 1983, Nature Conservancy Council: Peterborough, UK.

10. Hilton-Brown, D., Oldham, R.S., 1991. The Atatus of the Widespread Amphibians and Reptiles in Britain, 1990, and Changes during the 1980’s, Nature Conservancy Council Contract Survey 131. Nature Conservancy Council, Peterborough, UK.

11. UKBMS, 2014, UK Butterfly Monitoring Scheme: Summary of changes 2013, UKBMS,

12. Roy, H. et al 2011 Ladybirds (*Coccinellidae*) of Britain and Ireland, Field **Studies Council of behalf of the Centre for Ecology & Hydrology's Biological Records Centre**

**13.** Beckmann, B. C., B. V. Purse, D. B. Roy, H. E. Roy, P. G. Sutton, and C. D. Thomas. 2015. Two species with an unusual combination of traits dominate responses of British grasshoppers and crickets to environmental change. PloS one **10**:e0130488.

14. Cham, S., et al., Atlas of Dragonflies in Britain and Ireland. 2014, Telford: British Dragonfly Society.

15. Fox, R., et al., The State of Britain’s Larger Moths *2013*. 2013, Butterfly Conservation and Rothamsted Research: Wareham.

16. Fox, R., 2014a. The decline of moths in Great Britain: a review of possible causes. Insect Conservation and Diversity 6, 5-19.

17. Preston, C.D., D.A. Pearman, and T.D. Dines, New Atlas of British and Irish flora: An atlas of the vascular plants of Britain, Ireland, isle of man and the channel islands. 2002a, Oxford University Press: Oxford.

18. Carey, P.D., 2008. Results from 2007 Countryside Survey. CEH, Oxford.

19. UK NEA, 2011. The UK National Ecosystem Assessment. UNEP-WCMC, Cambridge.

20. DEFRA, *Biodiversity Indicators in Your Pocket*. 2013, DEFRA: London

21. Parry, H., et al, 2006. OBS 03: Quantitative approaches to assessment of farm level changes and implications for the environment, CSL & Countryside and Community Pearce-Higgins and Green 2014

22. Defra, 2014. Structure of the agricultural industry: Collection of statistical datasets. Department for Environment, Food & Rural Affairs, London, www.gov.uk/government/collections/structure-of-the-agricultural-industry.

23. Holmes, L. 2013. The British survey of Fertiliser Practice, Fertiliser use on farm crops for the crop year 2012. Defra, York.

24. Defra. 2014. Statistical data set, Agri-environment indicators. Department for Environment, Food & Rural Affairs, London, www.gov.uk/government/statistical-data-sets/agri-environment-indicators.

25. Hopkins, J. J., and K. J. Kirby. 2007. Ecological change in British broadleaved woodland since 1947. Ibis **149**:29-40.

26. Aebischer, N. J., Davey,P.D. & Kingdon,N.G. 2011. National Gamebag Census: Mammal Trends to 2009. Game & Wildlife Conservation Trust, Fordingbridge (http://www.gwct.org.uk/ngcmammals).

27. DECC. 2014. Collection: Renewables statistics - Renewable energy statistics collection covering the Digest of United Kingdom (DUKES), Energy Trends and regional data. Department of Energy & Climate Change, London: www.gov.uk/government/collections/renewables-statistics.

28. Mankelow, J. M., M. A. Sen, C. E. Wrighton, and N. Idoine. 2011. Collation of the results of the 2009 aggregate minerals survey for England and Wales. British Geological Survey, Department for Communities and Local Government, Welsh Assembly Government, London.

29. DfT. 2013. Collection: Road network size and condition - Road network size and condition statistical information. Department for Transport, London: https://www.gov.uk/government/collections/road-network-size-and-condition.

30. DfT. 2013. Collection: Road traffic statistics - Road traffic statistics information. Department for Transport, London: https://www.gov.uk/government/collections/road-traffic-statistics.

.31. Defra. 2014. Collection: Air quality and emissions statistics. Department for Environment, Food & Rural Affairs, London: <https://www.gov.uk/government/collections/air-quality-and-emissions-statistics>.

32. Fowler, D., Ashmore, M., Cape, N., Derwent, D., 2012. Review of transboundary air pollution (RoTAP): Acidification, Eutrophication, Ground Level Ozone and Heavy Metals in the UK. CEH, Penicuik.

33. DECC. 2013. Collection: Impacts of climate change in the UK. Department of Energy & Climate Change, London: https://www.gov.uk/government/collections/impacts-of-climate-change-in-the-uk.

34. Morecroft, M., Speakman, L. eds., 2013. Terrestrial Biodiversity Climate Change Impacts Summary Report. Living With Environmental Change.
